# Supplementary material for: Identifying the lifetime cognitive and socioeconomic antecedents of cognitive state: seven decades of follow-up in a British birth cohort study
Source: BMJ Open. 2019 Apr 24;9(4):e024404. doi: 10.1136/bmjopen-2018-024404 (PMC6502022; doi:10.1136/bmjopen-2018-024404)
Supplement: Supplementary data [file bmjopen-2018-024404supp001.pdf]

Supplementary Table 1 Associations between path variables and each ACE-III subscale: β (95% confidence intervals)

	Attention	Memory	Language	Fluency	Visuospatial
APOE ε4[1]	-0.05 (-0.1, 0.003)	-0.03 (-0.08, 0.01)	0.009 (-0.05, 0.06)	0.0003 (-0.04, 0.04)	0.003 (-0.05, 0.06)
Father's social class	-0.02 (-0.08, 0.03)	0.04 (-0.01, 0.09)	0.04 (-0.02, 0.09)	0.02 (-0.02, 0.05)*	0.04 (-0.02, 0.09)
Mother's education	0.04 (-0.01, 0.09)	0.01 (-0.03, 0.06)	-0.02 (-0.07, 0.03)	-0.007 (-0.04, 0.03)	-0.02 (-0.07, 0.04)
Childhood cognition	0.12 (0.05, 0.20)***	0.10 (0.04, 0.17)***	-0.01, -0.08, 0.06)	-0.09 (-0.13, -0.04)***	-0.008 (-0.08, 0.06)
Education	0.05 (-0.01, 0.12)	0.07 (0.02, 0.13)*	0.05 (-0.01, 0.12)	0.09 (0.05, 0.13)***	0.06 (-0.001, 0.13)
NART	0.04 (-0.03, 0.11)	0.30 (0.23, 0.36)***	0.06 (-0.02, 0.14)	0.19 (0.14, 0.25)***	0.04 (-0.04, 0.12)
Occupational complexity	0.01 (-0.04, 0.07)	0.05 (0.001, 0.10)*	0.06 (0.005, 0.12)*	0.08 (0.05, 0.12)***	0.06 (0.004, 0.12)*

[1] -0.05 (-0.09, -0.002), p = 0.04 for combined Attention and Memory

* p < 0.05, *** p < 0.001
