# Supplementary material for: Identifying the lifetime cognitive and socioeconomic antecedents of cognitive state: seven decades of follow-up in a British birth cohort study
Source: BMJ Open. 2019 Apr 24;9(4):e024404. doi: 10.1136/bmjopen-2018-024404 (PMC6502022; doi:10.1136/bmjopen-2018-024404)
Supplement: Author's manuscript [file bmjopen-2018-024404.draft_revisions.pdf]

BMJ Open is committed to open peer review. As part of this commitment we make the peer review history of every article we publish publicly available.

When an article is published we post the peer reviewers' comments and the authors' responses online. We also post the versions of the paper that were used during peer review. These are the versions that the peer review comments apply to.

The versions of the paper that follow are the versions that were submitted during the peer review process. They are not the versions of record or the final published versions. They should not be cited or distributed as the published version of this manuscript.

BMJ Open is an open access journal and the full, final, typeset and author-corrected version of record of the manuscript is available on our site with no access controls, subscription charges or pay-per-view fees (http://bmjopen.bmj.com).

If you have any questions on BMJ Open's open peer review process please email
info.bmjopen@bmj.com

BMJ Open

# Lifetime antecedents of cognitive state: seven decades of follow-up in a national birth cohort study

| | |
|---|---|
| Journal: | *BMJ Open* |
| Manuscript ID | bmjopen-2018-024404 |
| Article Type: | Research |
| Date Submitted by the Author: | 30-May-2018 |
| Complete List of Authors: | Richards, M; UCL, MRC Unit for Lifelong Health and Ageing at UCL<br>Stafford, Mai; UCL, MRC Unit for Lifelong Health and Ageing at UCL<br>Sharma, Nikhil; MRC Unit for Lifelong Health and Ageing; University College London Hospitals NHS Foundation Trust National Hospital for Neurology and Neurosurgery<br>Rawle, Mark; MRC Unit for Lifelong Health and Ageing<br>James, Sara Naomi; MRC Unit for Lifelong Health and Ageing<br>Davis, Daniel; MRC Unit for Lifelong Health and Ageing<br>Kuh, Diana; MRC Unit for Lifelong Health and Ageing, |
| Keywords: | EPIDEMIOLOGY, Dementia < NEUROLOGY, Old age psychiatry < PSYCHIATRY |
| | |

SCHOLARONE™
Manuscripts

ew only

Lifetime antecedents of cognitive state: seven decades of follow-up in a national birth cohort study

Marcus Richards [1], Mai Stafford [1], Nikhil Sharma [1,2], Mark Rawle [1], Sarah-Naomi James [1], Daniel Davis [1], Diana Kuh [1].

[1] MRC Unit for Lifelong Health and Ageing at UCL, 33 Bedford Place, London WC1B 5JU, UK

[2] National Hospital for Neurology and Neurosurgery, Queen Square, London WC1N 3BG, UK

**Correspondence to** Professor Marcus Richards, MRC Unit for Lifelong Health and Ageing at UCL, 33 Bedford Place, London WC1B 5JU, UK; m.richards@ucl.ac.uk tel. +44 (0)207 670 5710

Word count

Abstract: 221

Main text: 2813

Contributorship statement

MR conceived the work, conducted the statistical analyses and wrote the manuscript; MS advised on the path modelling; MS, NS, MR, SNJ, DD and DK contributed to interpretation and writing.

For peer review only

## ABSTRACT

**Objectives** The life course determinants of midlife and later life cognitive function have been studied using longitudinal population-based cohort data, but far less is known about whether the pattern of these pathways is similar or distinct for clinically-relevant cognitive state. We investigated this for the Addenbrooke's Cognitive Examination (ACE-III), used in clinical settings to screen for cognitive impairment and dementia.

**Methods** Based on the MRC National Survey of Health and Development (the British 1946 birth cohort), we used path modelling to test direct and indirect associations between APOE status, childhood and midlife socioeconomic position, childhood cognition, education, midlife verbal ability (National Adult Reading Test; NART), and the total ACE-III score.

**Results** *APOE* ε4 homozygosity showed a direct negative association with the ACE-III score, but not with prior cognition. Consistent with previous findings in this cohort for midlife cognition, the strongest influence on the ACE-III was from childhood cognition; and educational attainment was associated with the ACE-III independently of childhood cognition. The path from childhood cognition to the ACE-III was partly explained by the NART.

**Conclusions** The ACE-III in the general population shows a pattern of life course antecedents that is similar to neuropsychological measures of cognitive function, and may be utilised to represent normal cognitive ageing as well as a screen for cognitive impairment and dementia.

For peer review only

**Strengths and limitations of this study**

- The MRC National Survey of Health and Development (the British 1946 birth cohort) is the longest running study of its kind in the world, with a population-based sample and prospectively obtained information on socioeconomic status and educational attainment, and tested cognitive function from childhood
- Little is known about the life course antecedents of cognitive state, as determined by tests of mental status used to screen for cognitive impairment in research and clinical settings
- The Addenbrooke's Cognitive Examination (ACE-III) is the most comprehensive test of cognitive state available
- Path modelling used parameter estimates for incomplete data, thus minimising effects of missing predictor data
- The path structure of our model may be specific to cohort; NSHD is ethnically homogenous and experienced selective secondary education and high occupational mobility at labour market entry). Replication in more diverse populations is therefore required before our model can be considered generalisable

For peer review only

## INTRODUCTION

Using the MRC National Survey of Health and Development (NSHD; the British 1946 birth cohort)[1], we demonstrated multiple paths linking four fundamental developmental and social factors to midlife cognitive function: father's socioeconomic position (SEP), childhood cognitive ability, educational attainment, and own midlife SEP[2]. To our knowledge such a path model to understand key life course influences on cognitive state, as assessed in clinical practice, has not been undertaken. This is partly because the most frequently used tests, such as the Mini Mental Status Examination, are brief and have pronounced ceiling effects. It would however be valuable to investigate whether life course paths to cognitive state show a similar pattern as those for other cognitive functions, which would inform theoretical understanding of, and methodology for, studies of cognitive ageing across the full population range. At the most recent NSHD wave at age 69, the Addenbrooke's Cognitive Examination 3rd edition (ACE-III) was administered. This is the most extensive and comprehensive test of cognitive state available, with a quasi-normal distribution. Using this outcome we estimated a path model incorporating father's SEP, childhood cognitive ability, educational attainment and own midlife SEP, and adding two new paths. First, the National Adult Reading Test (NART), an outcome in the original path model, was now included as mediator; we hypothesised that influences on cognitive state operate significantly through this test. Second, the apolipoprotein E (*APOE*) gene was included, the best known genetic risk factor for dementia; based on previous work[3] we hypothesised that the ε4 allele of this gene would be negatively associated with the ACE-III score but not with childhood cognition.

For peer review only

## METHODS

### Participants

The NSHD is a representative sample of 5362 males and females born in England, Scotland, and Wales in one week in March 1946 (http://www.nshd.mrc.ac.uk/nshd). The 24th data collection was conducted between 2014 and 2015 when study members were aged 68-69 years[1]. At age 69 study members still alive and with a known current address in mainland Britain (n=2698) were invited to have a home visit by a trained nurse; 2149 (79.7%) completed a visit and a further 55 (2·0%) completed a postal questionnaire instead. Of the original cohort, 1026 (19·1%) had died, 578 (10·8%) were living abroad, 22 (0·4%) asked for their participation to be restricted to postal contacts, 621 (11·6%) had previously withdrawn from the study, and 417 (7·8%) had been lost to follow-up. For this data collection we obtained ethical approval from the NRES Queen Square REC (14/LO/1073), and Scotland A REC (14/SS/1009). All participants gave written informed consent to collect these data.

### Measures

Principal outcome: the ACE -III

The ACE-III is a screen-implemented test of cognitive state[4]. The ACE-III is divided into five domains: attention & orientation (scored 0-18), verbal fluency (0-14), memory (0-26), language (0-26), and visuospatial function (0-16). Thus the maximum total score is 100. Due to the inclusion of verbal fluency, the distribution of the total score is quasi-normal and avoids the pronounced ceiling effect of most cognitive state tests. A customised version of the ACE-III was administered by iPad using ACEMobile (http://www.acemobile.org/); where this was not possible, a paper version was used. All offline scoring was undertaken by trained personnel. Of the 2149 participants who had a home visit, 32 refused or were unable to undertake the ACE-III. Of the remaining 2117, 35 undertook but did not complete this; and

For peer review only

for the remaining 2082, data for 353 were lost through equipment failure. Thus complete ACE-III data were available for 1729 participants, 80·5% of those who received a home visit.

APOE genotype

Genetic data were assayed from a blood sample taken at age 53 by a research nurse. KBioscience then completed analysis of SNPs rs429358 and rs7412 which were used to determine APOE genotype. Distribution of alleles was as follows, ε2/ε2 n=20 (0·76%), ε2/ε3 n=307 (11·64%), ε3/ε3 n=1520 (57·64%), ε2/ε4 n=67 (2·54%), ε3/ε4 n=639 (24·23%), ε4/ε4 n=84 (3·19%). For analysis, APOE genotype was recoded categorically for the homozygous or heterozygous presence of ε4 alleles, with carriers of ε2 included as non *APOE* ε4 carriers. Because of difficulties in interpreting potentially opposing effects on cognition, the 67 participants with ε2/ε4 were excluded from analyses. Thus APOE was categorized as no ε4; heterozygous ε4; and homozygous ε4.

Choice of the following five path variables followed Richards & Sacker[2]:

Early life SEP

Early life SEP was assessed using the occupational social class of the father, classified when participants were aged 11 (or at 4 or 15 years if this was unknown) according to the UK Registrar General: professional, managerial, intermediate, skilled manual, semiskilled manual, unskilled. For comparability with the other variables these were coded so that higher values corresponded to higher position.

Childhood cognitive function

For peer review only

At 8 years participants took tests of verbal and nonverbal ability devised by the National Foundation for Educational Research[5], and administered by teachers or other trained personnel. These tests were: (1) reading comprehension (selecting appropriate words to complete 35 sentences), (2) word reading (ability to pronounce 50 words), (3) vocabulary (ability to explain the meaning of these 50 words), and (4) picture intelligence, consisting of a 60-item nonverbal reasoning test. Scores for each test were standardized to the whole sample, then summed to create a total score representing overall cognitive ability at this age.

Educational attainment

The highest educational or training qualification achieved by 26 years was grouped into no qualification, below ordinary secondary qualifications (vocational), ordinary secondary qualifications ('O' levels and their training equivalents), advanced secondary qualifications ('A' levels and their equivalents), or higher qualifications (degree or equivalent).

Adult SEP

Own current or last occupation by age 53 years was measured using similar categories to those for paternal occupation, and similarly coded so that higher values corresponded to higher position.

The NART

The NART assesses ability to pronounce 50 words of increasing difficulty[6]. These words violate conventional pronunciation rules, and are therefore unlikely to be read correctly unless the reader is familiar with them rather than relies on intelligent guesswork. Thus as a measure of 'crystallized' cognitive ability the NART is relatively insensitive to age and morbidity-associated decline, and serves as a measure of general cognitive ability. By

For peer review only

convention the NART is scored for errors, but for consistency with the childhood cognitive measure this was reverse-coded.

**Statistical methods**

All statistical analyses were conducted using Mplus version 5[7]. A preliminary multivariable linear regression model was used to test mutually adjusted associations between each of the predictor variables and the ACE-III total score. This incorporated full information maximum likelihood (FIML) parameter estimates to include those with item-missingness. FIML is preferable to estimation based on complete data, since FIML estimates tend to be less biased and more reliable than estimates based on list-wise deletion, even when the data deviate from missing at random and are non-ignorable[8]. However, each of these predictor variables are themselves closely related. Hence path modelling was then used to quantify their inter-associations independently of their associations with the ACE-III. We hypothesized two key components within this model: 1. strong paths from childhood cognition and the NART to the ACE-III, with modest and weak additional contributions from education and midlife SEP, respectively, and no direct path from childhood SEP[2]; 2. a direct negative path from APOE ε4 to the ACE-III but not via childhood cognition[3] or the NART. The path model also incorporated FIML. Three criteria were used to assess model fit: 1. the $\chi 2$ test, although this can be overly sensitive to model misspecification when sample sizes are large; 2. the root mean square error of approximation (RMSEA), which gives a measure of the discrepancy in fit per degrees of freedom. It is bounded below by zero, only taking this value if the model fits exactly. If the RMSEA is < 0.05, the model is considered a close fit to the data; 3. the comparative fit index (CFI), whose values are restricted to a 0 to 1 continuum, with higher values indicating a better fit. CFI is normally tested against a minimum criterion value of 0.95.

For peer review only

## RESULTS

### Descriptive

As noted, sample size was 1729, the maximum N for the ACE-III. Of those interviewed at age 69, there were no differences in any of the path variables between those with and without ACE-III data. Frequencies for each category of APOE group, childhood and midlife SEP and educational attainment, and means and SDs for the ACE-III and NART, are shown in Table 1.

For peer review only

**Table 1** Frequency distributions for APOE group, childhood and midlife SEP, educational attainment, and mean NART and ACE-III scores (for 1729 participants with ACE-III data)

| Variable | N | % | N | % |
|---|---|---|---|---|
| APOE | | | | |
| No ε4 | 1060 | (61·3) | | |
| Heterozygous ε4 | 369 | (21·3) | | |
| Homozygous ε4 | 47 | (2·7) | | |
| Missing | 253 | (14·6) | | |
| SEP | Childhood (father's) | | Midlife (own) | |
| Professional | 130 | (7·5) | 140 | (8·1) |
| Managerial | 362 | (20·9) | 687 | (39·7) |
| Intermediate | 289 | (16·7) | 418 | (24·2) |
| Skilled manual | 514 | (29·7) | 253 | (14·6) |
| Semiskilled | 267 | (15·4) | 168 | (9·7) |
| Unskilled | 77 | (4·5) | 52 | (3·0) |
| Missing | 90 | (5·2) | 11 | (0·6) |
| Educational attainment | | | | |
| No qualifications | 495 | (28·6) | | |
| Vocational only | 127 | (7·3) | | |
| Ordinary ('O') level | 354 | (20·5) | | |

For peer review only

| | | |
|---|---|---|
| Advanced ('A') level | 483 | (27·9) |
| Higher | 185 | (10·7) |
| Missing | 85 | (4·9) |
| | | |
| | Mean | (SD) |
| NART | 91.58 | (5.91) |
| ACE-III | 35.61 | (8.91) |

**Preliminary multivariable regression analyses**

Results for the preliminary multivariable linear regression analysis are shown in Table 2.

For peer review only

**Table 2** Standardized linear regression coefficients representing associations between gender, APOE status, childhood SEP, childhood cognition, educational attainment, midlife SEP and the NART, and the total ACE-III score at age 69 years (n=1729)

| Variable | β | 95% CI | p-value |
|---|---|---|---|
| Female gender | 0·04 | 0·00, 0·08 | .05 |
| APOE | | | |
| No ε4 (ref) | - | - | - |
| Heterozygous ε4 | -0·006 | -0·05, 0·04 | .77 |
| Homozygous ε4 | -0·04 | -0·09, -0·003 | .04 |
| Childhood SEP | 0·04 | -0·008, 0·08 | .11 |
| Childhood cognition | 0·18 | 0·12, 0·23 | <.0001 |
| Educational attainment | 0·10 | 0·05, 0·16 | <.0001 |
| Midlife SEP | 0·07 | 0·02, 0·12 | .003 |
| NART | 0·34 | 0·28, 0·40 | <.0001 |

*Note*. All coefficients are mutually adjusted, and represent change per point or level increase except for APOE, which are categorical. Abbreviations: β=standardized beta coefficient; CI=confidence intervals.

For peer review only

The strongest associations with the ACE-III were from prior cognition, above all the NART (standardized regression coefficient β=0·34 (0·28, 0·40)), then childhood cognition (β=0·18 (0·12, 0·23)). Educational attainment showed a modest association (β=0·10 (0·05, 0·16)), with midlife SEP somewhat weaker (β=0·07 (0·02, 0·12)). There was no independent association with childhood SEP (β=0·04 (-0·01, 0·08). When compared with absence of APOE ε4, ε4 homozygosity was weakly negatively associated with the ACE-III score (β=-0·04 (-0·09, -0·003)), but not heterozygosity (β=-0·01 (-0·05, 0·04)). Female gender was weakly positively associated with this score (β=0·04 (0, 0·08)).

**Path model**

Figure 1 shows the path model. All paths are mutually adjusted, and, following the regression model, paths were adjusted for gender. Goodness of fit statistics indicated that the model was an adequate representation of the data ($\chi^2$=7·96, *df*=6, p=0·24; RMSEA=0.01, 95% CI=0-0·04, p=1·0; CFI = 1·0). All non-significant paths (*P* value >.05) were removed in the final model shown.

[Figure 1 about here]

The strongest influences on the ACE-III score were from the NART, and from childhood cognition, which was mainly associated with the ACE-III via educational attainment and the NART, but also directly with the ACE-III. The influence of midlife SEP was more modest, and was itself part-mediated by the NART. There was no direct path from childhood SEP to the ACE-III, but childhood SEP had independent associations with childhood cognition, educational attainment and midlife SEP, in descending order of magnitude. APOE ε4 homozygosity showed a modest direct negative association with the ACE-III score, but was not associated with childhood cognition or the NART; and ε4 heterozygosity was not associated with any cognitive variable.

For peer review only

**DISCUSSION**

In the NSHD we estimated a path model describing key life course influences on cognitive state using the Addenbrooke's cognitive examination (ACE-III). Confirming our main study hypothesis, by far the strongest influence on this outcome was from lifetime cognition, most strongly from general cognitive ability in midlife, assessed by the NART. The NART in turn was particularly influenced by childhood cognition. To a lesser extent educational attainment was positively associated with the ACE-III, independently of childhood cognition, although the model suggests that this was part-mediated by the NART. Own SEP showed more modest effects still, and there was no direct association between childhood SEP and the ACE-III. Finally, there was a direct negative association between the *APOE* ε4 allele and the ACE-III; ε4 was not associated with childhood cognitive function, or with the NART. The pattern of associations for SEP, childhood cognition and education broadly reflect those previously shown in this cohort when the NART was an outcome rather than a predictor[2], even with an important genetic influence on cognitive function (APOE ε4) controlled. However, it is notable that, with the NART controlled, childhood cognition, education and midlife SEP additionally showed direct associations with the ACE-III, with childhood cognition having the strongest effect, and midlife SEP the weakest.

Major strengths of this study are: 1. the use of a large representative population-based birth cohort; 2. the most extensive and comprehensive measure of cognitive state (ACE-III) available as an outcome; 3. prospective measures across the life course, including tested childhood cognition, which enabled the first comprehensive prospective life course model of mental state; 4. path modelling that uses FIML parameter estimates for incomplete data, thus minimizing effects of missing predictor data. Against these strengths we should note that the path structure of our model may be specific to cohort (NSHD is ethnically homogenous) and period (NSHD experienced selective secondary education and high occupational mobility at

labor market entry). While our previous work suggests a broadly robust path structure in the face of social change[9], replication in more diverse populations is required before our model can be considered generalizable.

Our path model suggests that cognitive state has a prominent general cognitive ability component, which in turn has cognitive antecedents extending back into childhood. It might be argued that the influence of the NART is a matter of circularity, reflecting the dominance of verbal-based tests within the ACE-III (accounting for 84% of the total score). However, the NART also correlates with non-verbal skills[5]. The most obvious difference between the NART and the ACE-III is that the constituent tests of the latter are ‘fluid’ measures, sensitive to age and morbidity-associated decline; whereas the former, as a measure of ‘crystallized’ ability, is stable even in the face of mild dementia[10]. Further follow-up will determine whether the cognitive paths within our model retain their magnitude and pattern as the ACE-III scores change over time.

In regard to the long-term cognitive antecedents of the ACE-III, the present study extends our previous studies showing that childhood cognition tracks across the life course even when education, lifetime socioeconomic position[2] and adolescent mental health[11] are controlled. This tracking is also consistent with earlier studies in relation to cognitive ageing[12] and risk of dementia[13,14]; and with studies showing that associations between tests of mental state and verbal cognitive ability are strongly explained by childhood cognitive function[15,16]. We also observed an additional direct association between childhood cognition and the ACE-III that was independent of the NART as well as other factors in the model. This is probably because the measures of childhood cognition capture a wider range of function than the NART, including nonverbal reasoning, even though, as noted, the NART itself predicts a comprehensive range of cognitive function[5].

For peer review only

The next most prominent influence on the ACE-III was from educational attainment. This was associated with the ACE-III even when childhood cognition was controlled. As with childhood cognition itself, the influence of education was largely through the NART, although again there was a modest independent association with the ACE-III, since education also shapes non-verbal cognitive skills[17]. An association between education and subsequent cognition independent of childhood cognition has long been observed[18]; has been replicated in two other birth cohorts[19]; is shown in NSHD to be additive with respect to adult education[20]; and responds rapidly to policy[9]. By way of interpretation, it is important to note that schooling is not just a process of 'cognitive stimulation'. Schools indeed teach specific knowledge, but can also teach practical skills, including how to approach cognitive testing, refine other cognitive skills, and shape non-cognitive skills that are likely to have long-term benefit to cognitive function[21,11]. Policies to improve access to education, and widen educational curricula to strengthen all these skills, are likely to have long-term benefits to cognitive ageing, and risk of dementia.

Finally, we should consider the role of APOE in the model. This is involved in the transport of cholesterol and other lipids between cellular structures, and ε4 has a higher rate of lipoprotein clearance thus altering its bioavailability[22]. APOE is also involved in clearing beta amyloid from the brain, and ε4 may be less efficient at this[23]. A direct association between the ε4 allele and the ACE-III was found in our model; this was of relatively weak magnitude, was only observed in homozygotes, and was not observed with any other variable in the model including prior cognitive function. These findings are consistent with evidence that ε4 zygosity shows a dose-response for Alzheimer's disease[24]; with a study showing no association with childhood cognition although observed in old age in the same cohort[3]; and with parallel evidence from NSHD that decline in verbal memory from age 43 to 69 is faster in APOE homozygosity[25]. There is no consensus over whether APOE is associated with

For peer review only

normal cognitive ageing as opposed to clinical decline[3,24,25-30]. However, this may be age-dependent[27]; intriguingly, while no association was found between ε4 and fluid cognitive measures in NSHD at age 53[28], this association is now evident 16 years later, albeit modestly. It should also be highlighted that the presence of APOE in the model means that the structure and magnitude of the pathways, including those between parental social class and childhood cognition, were independent of this. Adding APOE does not of course comprehensively control for genetic influence on cognitive ageing. However, the ε4 allele of this gene is the best-known genetic risk factor for clinically significant cognitive decline[31].

In conclusion, the ACE-III in the general ageing population shows a pattern of life course antecedents that is similar to neuropsychological measures of cognitive function. This may not have emerged from studies using briefer tests of cognitive state such as the MMSE, since most of these have ceiling effects outside the clinical context that limit their use as continuous measures. As noted, continuing follow-up of NSHD will elucidate whether the path structure we describe here changes as an increasing number of participants meet clinical criteria for dementia, and the distribution of the ACE-III shifts accordingly.

For peer review only

**Acknowledgements**

We thank NSHD study members for their lifelong participation and past and present members of the NSHD study team who helped to collect the data. We also thank ACE Mobile for providing a customized version of the ACE-III for NSHD.

**Funding**

This study was supported by the UK Medical Research Council, which provides core funding for the NSHD, and supports the authors by MC_UU_12019/1 (Enhancing NSHD), and /3 (Mental Ageing). DD is additionally funded through a Wellcome Trust Intermediate Clinical Fellowship (WT107467). SNJ is additionally funded by Alzheimer's Research UK (ARUK-PG2014–1946, ARUK-PG2017-1946).

**Data Sharing**

Bona fide researchers can apply to access the NSHD data via a standard application procedure (further details available at: http://www.nshd.mr. cac.uk/data.aspx).

**Patient and Public Involvement**

Participants have a lifelong association with NSHD. Over the 70 years of the study, the research team has increasingly involved participants, in line with changing norms about conducting cohort studies, starting at age 16 (in 1962) with the annual dissemination of study findings in birthday cards and this continues. Participants have always received a personal letter from the Director whenever they have raised queries or provided additional comments, including suggestions for new topics to study. In the last ten years, the research team has increased the level of participant involvement through invitations to study events and focus groups to discuss clinical sub-studies; and a new participant website (www.nshd.mrc.ac.uk/study-members/) was developed in line with their feedback.  When

For peer review only

piloting new questionnaires and assessments, we recruit patients from GP practices or from the UCLH PPI and take into account their feedback when designing the mainstage fieldwork for NSHD participants.

**Competing interests**

None disclosed.

Figure legend: Figure 1. Path model for the ACE-III total score

For peer review only

**REFERENCES**

1 Kuh D, Wong A, Shah I, et al. The MRC National Survey of Health and Development reaches age 70: maintaining participation at older ages in a birth cohort study. *Eur J Epidemiol* 2016;31:1135–1147.

2 Richards M, Sacker A. Lifetime antecedents of cognitive reserve. *J Clin Exp Neuropsychol* 2003;25:614–624.

3 Deary IJ, Whiteman MC, Pattie A, et al. Cognitive change and the APOE epsilon 4 allele. *Nature* 2002;418:932. (erratum Nature 2002;419:450)

4 Hsieh S, Schubert S, Hoon C, et al. Validation of the Addenbrooke's Cognitive Examination III in frontotemporal dementia and Alzheimer's disease. *Dement Geriatr Cogn Disord* 2013;36:242–250.

5 Pigeon DA. Tests used in the 1954 and 1957 surveys. In: Douglas JWB ed. The home and the school. London: Macgibbon & Key 1964 appendix 1:129-132.

6 Nelson HE, Willison JR. National Adult Reading Test (NART). 2nd ed. Windsor: NFER-Nelson 1991.

7 Muthén LK, Muthén BO. Mplus. Statistical analysis with latent variables. User's guide. Los Angeles, CA: Muthén and Muthén 2004.

8 Arbuckle JC. Full information estimation in the presence of incomplete data. In: Marcoulides GA, Schumacker RE eds. Advanced Structural Equation Modeling Techniques. Mahwah, NJ: Lawrence Erlbaum Associates, 1996:243-277.

9 Richards M, Power C, Sacker A. Paths to literacy and numeracy problems: evidence from two British birth cohorts. *J Epidemiol Community Health* 2009;63:239–244.

10 McGurn B, Starr JM, Topfer JA, et al. Pronunciation of irregular words is preserved in dementia, validating premorbid IQ estimation. *Neurology* 2004;62:1184–1186.

For peer review only

11 Xu MK, Jones PB, Barnett JH, et al. Adolescent self-organization predicts midlife memory in a prospective birth cohort study. *Psychology Aging* 2013;28:958–68.

12 Plassman B, Welsh K, Helms M, et al. Intelligence and education as predictors of cognitive state in late life: A 50-year follow-up. *Neurology* 1995;45:1446–1450.

13 Snowdon DA, Kemper SJ, Mortimer JA, et al. Linguistic ability in early life and cognitive function and Alzheimer's disease in late life. Findings from the Nun Study. *JAMA* 1996;275:528–532.

14 McGurn B, Deary IJ, Starr JM. Childhood cognitive ability and risk of late-onset Alzheimer and vascular dementia. *Neurology* 2008;71:1051–1056.

15 Crawford JR, Deary IJ, Starr J, et al. The NART as an index of prior intellectual functioning: a retrospective validity study covering a 66-year interval. *Psychol Med* 2001;31:451–458.

16 Dykiert D, Der G, Starr JM, et al. Why is Mini-Mental state examination performance correlated with estimated premorbid cognitive ability? *Psychol Med* 2016;46:2647–2654.

17 Roselli R, Ardila A. The impact of culture and education on non-verbal neuropsychological measurements: A critical review. *Brain Cognition* 2003;52:326–333.

18 Snow RE, Yalow E. Education and intelligence. In: Sternberg RJ, ed. Handbook of human intelligence. Cambridge, MA: Cambridge University Press, 1982:493-585.

19 Clouston S, Kuh D, Herd P, et al. Benefits of educational attainment on adult fluid cognition: international evidence from three birth cohorts. *Int J Epidemiol* 2012;41:1729–1736.

For peer review only

20 Hatch SL, Feinstein L, Link B, et al. The continuing benefits of education: adult education and midlife cognitive ability in the British 1946 birth cohort. *J Gerontol Series B* 2007;62:S404–414.

21 Richards M, Hatch, SL. A life course approach to the development of mental skills. *Journal of Gerontology Series B* 2011;66:Suppl 1, i26–35.

22 Poirier J, Davignon J, Bouthillier D, et al. Apolipoprotein E polymorphism and Alzheimer's disease. *Lancet* 1993;342:697–699.

23 Jiang Q, Lee CY, Mandrekar S, et al. ApoE promotes the proteolytic degradation of Aβ. *Neuron* 2008;58:681–693.

24 Jorm AF, Mather KA, Butterworth P, et al. APOE genotype and cognitive functioning in a large age-stratified population sample. *Neuropsychology* 2007;21:1–8.

25 Rawle M, Davis D, Bendayan B, et al. Apolipoprotein-E (APOE) ε4 and cognitive decline over the adult life course. *Transl Psychiat* 2018 Jan 10;8:18. doi: 10.1038/s41398-017-0064-8.

26 Caselli RJ, Dueck AC, Osborne D, et al. Longitudinal Modeling of Age-Related Memory Decline and the APOE ε4 Effect. *N Engl J Med* 2009;361:255–263.

27 Wisdom NM, Callahan JL, Hawkins KA. The effects of apolipoprotein E on non-impaired cognitive functioning: a meta-analysis. *Neurobiol Aging* 2011;32:63–74.

28 Alfred T, Ben-Shlomo Y, Cooper R, et al. Associations between APOE and low-density lipoprotein cholesterol genotypes and cognitive and physical capability: the HALCyon programme. *Age* 2014;36:9673.

29 Bunce D, Bielak AA, Anstey KJ, et al. APOE genotype and cognitive change in young, middle-aged, and older adults living in the community. *J Gerontol A Biol Sci Med Sci* 2014;69:379–386.

For peer review only

30 Knight RG, Tsui HS, Abraham WC, et al. Lack of effect of the apolipoprotein E epsilon4 genotype on cognition during healthy aging. *J Clin Exp Neuropsychol* 2014:36:742–750.

31 Farrer LA, Cupples LA, Haines JL, et al. Effects of age, sex, and ethnicity on the association between apolipoprotein E genotype and Alzheimer disease. A meta-analysis. APOE and Alzheimer Disease Meta Analysis Consortium. *JAMA* 1997;278:1349–1356.

For peer review only

For peer review only

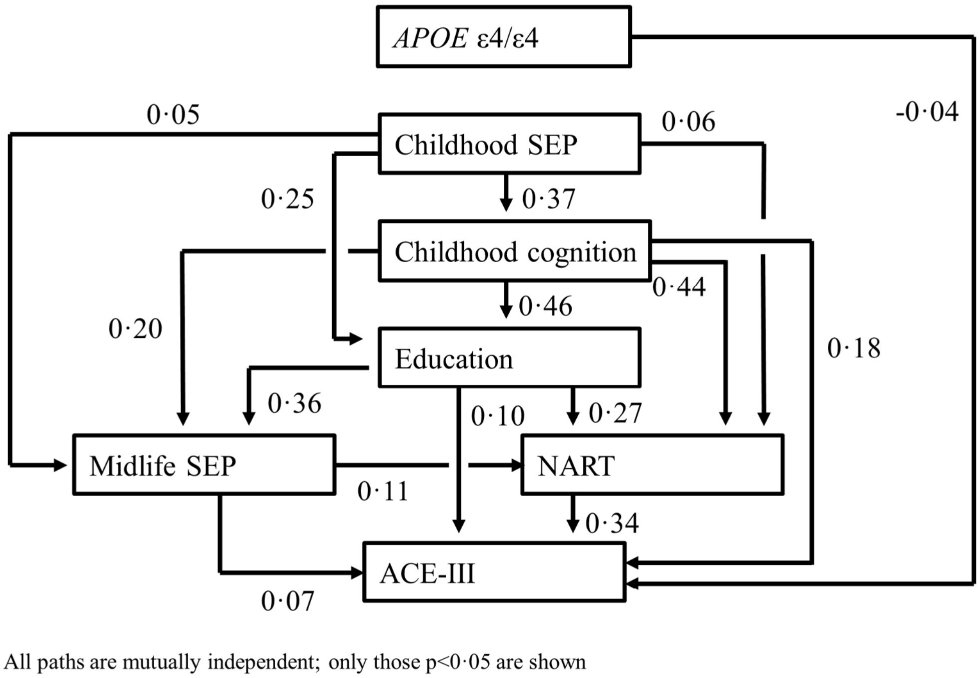


Path model for the ACE-III total score

170x119mm (300 x 300 DPI)

BMJ Open

# Identifying the lifetime cognitive and socioeconomic antecedents of cognitive state: seven decades of follow-up in a British birth cohort study.

| | |
|---|---|
| Journal: | *BMJ Open* |
| Manuscript ID | bmjopen-2018-024404.R1 |
| Article Type: | Research |
| Date Submitted by the Author: | 04-Oct-2018 |
| Complete List of Authors: | Richards, M; UCL, MRC Unit for Lifelong Health and Ageing at UCL<br>James, Sarah-Naomi; MRC Unit for Lifelong Health and Ageing<br>Sizer, Alison; University College London, Epidemiology and Public Health<br>Sharma, Nikhil; MRC Unit for Lifelong Health and Ageing; University College London Hospitals NHS Foundation Trust National Hospital for Neurology and Neurosurgery<br>Rawle, Mark; MRC Unit for Lifelong Health and Ageing<br>Davis, Daniel; MRC Unit for Lifelong Health and Ageing<br>Kuh, Diana; MRC Unit for Lifelong Health and Ageing, |
| <b>Primary Subject Heading</b>: | Epidemiology |
| Secondary Subject Heading: | Geriatric medicine |
| Keywords: | EPIDEMIOLOGY, Dementia < NEUROLOGY, Old age psychiatry < PSYCHIATRY |
| | |

SCHOLARONE™
Manuscripts

Identifying the lifetime cognitive and socioeconomic antecedents of cognitive state: seven decades of follow-up in a British birth cohort study

Marcus Richards [1], Sarah-Naomi James [1], Alison Sizer [2], Nikhil Sharma [1,3], Mark James Rawle [1], Daniel Davis [1], Diana Kuh [1].

[1] MRC Unit for Lifelong Health and Ageing at UCL, 33 Bedford Place, London WC1B 5JU, UK

[2] Department of Epidemiology and Public Health, University College London, 1-19 Torrington Place, London, WC1E 7HB, UK

[3] National Hospital for Neurology and Neurosurgery, Queen Square, London WC1N 3BG, UK

**Correspondence to** Professor Marcus Richards, MRC Unit for Lifelong Health and Ageing at UCL, 33 Bedford Place, London WC1B 5JU, UK; m.richards@ucl.ac.uk tel. +44 (0)207 670 5710

For peer review only

Word count

Abstract: 266

Main text: 3509

Contributorship statement

MR conceived the work, conducted the statistical analyses and wrote the manuscript; SNJ advised on the path modelling; SNJ, AS, NS, MJR,, DD and DK contributed to interpretation and writing.

## ABSTRACT

**Objectives** The life course determinants of midlife and later life cognitive function have been studied using longitudinal population-based cohort data, but far less is known about whether the pattern of these pathways is similar or distinct for clinically-relevant cognitive state. We investigated this for the Addenbrooke's Cognitive Examination (ACE-III), used in clinical settings to screen for cognitive impairment and dementia.

**Design** Longitudinal birth cohort study.

**Setting** Residential addresses in England, Wales and Scotland.

**Participants** 1762 community-dwelling men and women of European heritage, enrolled since birth in the MRC National Survey of Health and Development (the British 1946 birth cohort).

**Primary outcome** The Addenbrooke's Cognitive Examination (ACE-III).

**Results** Path modelling estimated direct and indirect associations between *APOE* status, father's social class, childhood cognition, education, midlife occupational complexity, midlife verbal ability (National Adult Reading Test; NART), and the total ACE-III score. Controlling for sex, there was a direct negative association between *APOE* ε4 and the ACE-III score (β=-0.04, [-0.08, -0.002], p=0.04), but not between *APOE* ε4 and childhood cognition (β=0.03 [-0.006, 0.69, p=0.10] or the NART (β=0.0005 [-0.03, 0.03], p=0.97). The strongest influences on the ACE-III were from childhood cognition (β=0.20 [0.14, 0.26], p<0.001) and the NART (β=0.35 [0.29, 0.41], p<0.001); educational attainment and

For peer review only

occupational complexity were modestly and independently associated with the ACE-III ($\beta$=0.08 [0.03, 0.14], p=0.002 and $\beta$=0.05 [0.01, 0.10], p=0.02, respectively).

**Conclusions** The ACE-III in the general population shows a pattern of life course antecedents that is similar to neuropsychological measures of cognitive function, and may be utilised to represent normal cognitive ageing as well as a screen for cognitive impairment and dementia.

For peer review only

**Strengths and limitations of this study**

- The MRC National Survey of Health and Development (the British 1946 birth cohort) is a large population-based sample with prospectively obtained information on socioeconomic status and educational attainment, and tested cognitive function from childhood
- The Addenbrooke's Cognitive Examination (ACE-III) is an extensive and comprehensive test of cognitive state.
- Path modelling used parameter estimates for incomplete data, thus minimising effects of missing predictor data
- The path structure of our model may be specific to cohort; NSHD is ethnically homogenous and experienced selective secondary education and high occupational mobility at labour market entry).
- Replication in more diverse populations is therefore required before our model can be considered generalisable

For peer review only

## INTRODUCTION

Using the MRC National Survey of Health and Development (NSHD; the British 1946 birth cohort)[1], we demonstrated multiple paths linking four fundamental developmental and social factors to midlife cognitive function: father's socioeconomic position (SEP), childhood cognitive ability, educational attainment, and own midlife SEP[2]. To our knowledge such a path model to understand key life course influences on cognitive state, as assessed in clinical practice, has not been undertaken. This is partly because the most frequently used tests, such as the Mini Mental Status Examination (MMSE) and Montreal Cognitive Assessment (MoCA), are brief and have pronounced ceiling effects. It would however be valuable to investigate whether life course paths to cognitive state show a similar pattern as those for other cognitive functions, which would inform theoretical understanding of, and methodology for, studies of cognitive ageing across the full population range. At the most recent NSHD wave at age 69, the Addenbrooke's Cognitive Examination 3rd edition (ACE-III) was administered. This is a more extensive and comprehensive test of cognitive state than the MMSE or MoCA, with a quasi-normal distribution. Using this outcome we estimated a path model incorporating childhood SEP, childhood cognitive ability, educational attainment and midlife occupational complexity, and adding two new paths. First, the National Adult Reading Test (NART), an outcome in the original path model, was now included as an intervening variable; we hypothesised that influences on cognitive state operate significantly through this test. Second, the apolipoprotein E (*APOE*) gene was included, the best known genetic risk factor for dementia; based on previous work[3] we hypothesised that the ε4 allele of this gene would be negatively associated with the ACE-III score but not with childhood cognition.

For peer review only

## METHODS

### Participants

The NSHD is a representative sample of 5362 males and females born in England, Scotland, and Wales in one week in March 1946 (http://www.nshd.mrc.ac.uk/nshd). The 24th data collection was conducted between 2014 and 2015 when study members were aged 68-69 years[1]. At age 69 study members still alive and with a known current address in mainland Britain (n=2698) were invited to have a home visit by a trained nurse; 2149 (79.7%) completed a visit and a further 55 (2·0%) completed a postal questionnaire instead. Of the original cohort, 1026 (19·1%) had died, 578 (10·8%) were living abroad, 22 (0·4%) asked for their participation to be restricted to postal contacts, 621 (11·6%) had previously withdrawn from the study, and 417 (7·8%) had been lost to follow-up. For this data collection we obtained ethical approval from the NRES Queen Square REC (14/LO/1073), and Scotland A REC (14/SS/1009). All participants gave written informed consent to collect these data.

### Measures

Principal outcome: the ACE -III

The ACE-III is a screen-implemented test of cognitive state, and has been validated as a screening tool for cognitive deficits in Alzheimer's disease and frontotemporal dementia[4]. The ACE-III is divided into five domains: attention & orientation (scored 0-18), verbal fluency (0-14), memory (0-26), language (0-26), and visuospatial function (0-16). Thus the maximum total score is 100. Due to the inclusion of verbal fluency, the distribution of the total score is quasi-normal and avoids the pronounced ceiling effect of most cognitive state tests. A customised version of the ACE-III was administered by iPad using ACEMobile (http://www.acemobile.org/); where this was not possible, a paper version was used. All offline scoring was undertaken by trained personnel. Of the 2149 participants who had a

For peer review only

home visit, 32 refused or were unable to undertake the ACE-III. Of the remaining 2117, 35 undertook but did not complete this; and for the remaining 2082, data for 320 were lost through equipment failure. Thus complete ACE-III data were available for 1762 participants, 82·0% of those who received a home visit.

Genetic risk

Genetic risk was primarily represented by the *APOE* ε4 allele. Using blood taken at age 53 or 69-71 by a research nurse, KBioscience analysed SNPs rs429358 and rs7412 to determine *APOE* genotype. Distribution of alleles was as follows (n = 2686), ε2/ε2 n=20 (0·74%), ε2/ε3 n=318 (11·84%), ε3/ε3 n=1538 (57·26%), ε2/ε4 n=68 (2·53%), ε3/ε4 n=657 (24·46%), ε4/ε4 n=85 (3·16%). For analysis, *APOE* genotype was recoded categorically for the presence of ε4 alleles, with carriers of ε2 included as non *APOE* ε4 carriers. Because of difficulties in interpreting potentially opposing effects on cognition, the 68 participants with ε2/ε4 were excluded. Thus *APOE* was categorized as no ε4 vs. heterozygous ε4 or homozygous ε4. For comparison with *APOE*, polygenic scores (PGS) for Alzheimer's disease were calculated for 2,768 participants using blood samples taken at age 53 and 60-64. Genotyping was carried out on the NeuroX2 chip. PGSs were created using Allelic Scoring function in PLINK_v1.9. The base dataset used to calculate the PGS was the large, two-stage meta-analysis of genome-wide association studies in individuals of European heritage conducted by the International Genomics of Alzheimer's Project (IGAP)[5]. Linkage-disequilibrium parameters were set to r2>0.2 and a physical distance threshold for clumping SNPs set to 1Mb. The PGS included the SNPs with a p-value in the I-GAP meta-analysis of p<0.05 (n= 31746).

Early life SEP

Early life SEP was assessed using father's occupational social class and mother's education, which is associated with offspring cognition independently of father's occupation[6]. The

For peer review only

former was classified when participants were aged 11 (or at 4 or 15 years if this was unknown) according to the UK Registrar General: professional, managerial, intermediate, skilled manual, semiskilled manual, unskilled; mother's education was coded as primary only vs. secondary or any formal qualifications.

Childhood cognitive function

At 8 years participants took tests of verbal and nonverbal ability devised by the National Foundation for Educational Research[7], and administered by teachers or other trained personnel. These tests were: (1) reading comprehension (selecting appropriate words to complete 35 sentences), (2) word reading (ability to pronounce 50 words), (3) vocabulary (ability to explain the meaning of these 50 words), and (4) picture intelligence, consisting of a 60-item nonverbal reasoning test. Scores for each test were standardized to the whole sample, then summed to create a total score representing overall cognitive ability at this age.

Educational attainment

The highest educational qualification achieved by 43 years was grouped into no qualification, below ordinary secondary qualifications (vocational), ordinary secondary qualifications ('O' levels and their training equivalents), advanced secondary qualifications ('A' levels and their equivalents), or higher qualifications (degree or equivalent).

Midlife occupational complexity

Midlife occupational complexity was represented by the National Statistics Socio-Economic Classification (NS-SEC) of the job held at age 53 or earlier if this was missing[8]. This provides a measure of employment relations and the conditions of employment, based on the Standard Occupational Classification (SOC): details of individual employment status (i.e.

For peer review only

employer/ employee/ self-employed); supervisory position; and number of employees in the workplace. For NSHD information was available on the start and end dates of up to 27 jobs and their NS-SEC categories, which were recoded into 7 classes: 1. Higher managerial, administrative and professional occupations; 2. Lower managerial, administrative and professional occupations; 3. Intermediate occupations; 4. Small employers and own account workers; 5. Lower supervisory and technical occupations; 6. Semi-routine occupations; 7. Routine occupations.

The NART

The NART assesses ability to pronounce 50 words of increasing difficulty[9]. These words violate conventional pronunciation rules, and are therefore unlikely to be read correctly unless the reader is familiar with them rather than relies on intelligent guesswork. Thus the NART serves as a measure of general (crystallised') cognitive ability.

All measures were coded so that higher values signified higher status or function.

**Statistical methods**

All statistical analyses were conducted using STATA version 15[10]. Path modelling was used to quantify associations between each predictor variable and the ACE-III. Since each of the predictor variables are closely inter-related, the model also quantifed their independent inter-associations. We hypothesized two key components within this model: 1. strong paths from childhood cognition and the NART to the ACE-III, with modest and weak additional contributions from education and midlife occupational complexity, respectively, and no direct path from childhood SEP[2]; 2. a direct negative path from APOE ε4 to the ACE-III but not via childhood cognition[3] or the NART. No directionality of association was

For peer review only

assumed between mother's education and father's social class, or between occupational complexity and the NART; these paths are therefore represented as correlational only. The path model was adjusted for gender, andincorporated full information maximum likelihood (FIML) parameter estimates to include those with item-missingness. FIML is preferable to estimation based on complete data, since FIML estimates tend to be less biased and more reliable than estimates based on list-wise deletion, even when the data deviate from missing at random and are non-ignorable[11]. Three criteria were used to assess model fit: 1. the $\chi 2$ test, although this can be overly sensitive to model misspecification when sample sizes are large; 2. the root mean square error of approximation (RMSEA), which gives a measure of the discrepancy in fit per degrees of freedom. It is bounded below by zero, only taking this value if the model fits exactly. If the RMSEA is < 0.05, the model is considered a close fit to the data; 3. the comparative fit index (CFI), whose values are restricted to a 0 to 1 continuum, with higher values indicating a better fit. CFI is normally tested against a minimum criterion value of 0.95.

**Patient and Public Involvement**

Participants have a lifelong association with NSHD. Over the 70 years of the study, the research team has increasingly involved participants, in line with changing norms about conducting cohort studies, starting at age 16 (in 1962) with the annual dissemination of study findings in birthday cards and this continues. Participants have always received a personal letter from the Director whenever they have raised queries or provided additional comments, including suggestions for new topics to study. In the last ten years, the research team has increased the level of participant involvement through invitations to study events and focus groups to discuss clinical sub-studies; and a new participant website (www.nshd.mrc.ac.uk/study-members/) was developed in line with their feedback. When

For peer review only

piloting new questionnaires and assessments, we recruit patients from GP practices or from the UCLH PPI and take into account their feedback when designing the mainstage fieldwork for NSHD participants.

## RESULTS

### Descriptive

As noted, sample size was 1762, the maximum N for the ACE-III. Those who were not interviewed at age 69 for any reason showed no difference in *APOE* ε4 frequency (p=0.72) but had lower childhood cognition and NART scores, and were more likely to be disadvantaged in terms of father's social class, mother's education, own education and occupational complexity (all p<0.001). Those not interviewed were also previously shown to have three or more clinical disorders at the previous assessment (age 60-64), a general health self-rating as poor or fair rather than good, and a longstanding limiting illness, although the latter was not associated with interview participation after controlling for socioeconomic and cognitive characteristics[1]. Of those interviewed at age 69, there were no differences in any of the path variables between those with and without ACE-III data, except for a slight trend for the ACE-III to be missing in those with no educational qualifications ($\chi 2=9.5$, p= 0.05). Frequencies for each category of APOE group, childhood and midlife SEP and educational attainment, and means and SDs for the ACE-III and NART, are shown in Table 1.

**Table 1** Frequency distributions for APOE group, childhood and midlife SEP, educational attainment, and mean NART and ACE-III scores (for 1762 participants with ACE-III data)

| Variable | N | % | N | % |
|---|---|---|---|---|

For peer review only

| | | |
|---|---|---|
| *APOE* | | |
| No ε4 | 1076 | (61·1) |
| Heterozygous ε4 | 388 | (21·0) |
| Homozygous ε4 | 48 | (2·7) |
| Missing | 250 | (14·2) |
| Father's social class | | |
| Professional | 134 | (7·6) |
| Managerial | 372 | (21·1) |
| Intermediate | 296 | (16·8) |
| Skilled manual | 519 | (29·4) |
| Semiskilled | 271 | (15·4) |
| Unskilled | 79 | (4·5) |
| Missing | 91 | (5·2) |
| Mother's education | | |
| Primary only | 1151 | (65.3) |
| Secondary or any formal qualifications | 421 | (23.9) |
| Missing | 611 | (10.8) |
| Educational attainment (by age 43) | | |
| No qualifications | 403 | (22.9) |
| Vocational only | 235 | (13·3) |

For peer review only

| | | |
|---|---|---|
| Ordinary ('O') level | 340 | (19.3) |
| Advanced ('A') level | 497 | (28.2) |
| Higher | 2.73 | (15.5) |
| Missing | 14 | (0.8) |
| | | |
| NSSEC occupation (by age 53)* | | |
| 1. | 221 | (12.5) |
| 2. | 504 | (28.6) |
| 3. | 307 | (17.4) |
| 4. | 200 | (11.4) |
| 5. | 120 | (6.8) |
| 6. | 228 | (12.9) |
| 7. | 154 | (8.7) |
| Missing | 28 | (1.6) |
| | | |
| | Mean | (SE) |
| NART | 35.6 | (0.22) |
| ACE-III | 91.52 | (0.14) |

*1. Higher managerial, administrative and professional occupations; 2. Lower managerial, administrative and professional occupations; 3. Intermediate occupations; 4. Small employers and own account workers; 5. Lower supervisory and technical occupations; 6. Semi-routine occupations; 7. Routine occupations.

For peer review only

**Path model**

Figure 1 shows the path model. All paths are mutually adjusted. Goodness of fit statistics indicated that the model was an excellent representation of the data ($\chi^2$=0.15, p=1.0 for analytic vs. saturated model; RMSEA=0, p=1·0; CFI = 1·0). Gender effects and all non-significant paths (*P* value >.05) are not shown.

[Figure 1 about here]

The strongest influences on the ACE-III score were from the NART, and from childhood cognition, which was mainly associated with the ACE-III via educational attainment and the NART, but also directly with the ACE-III. The influence of midlife occupational complexity was more modest, and was itself part-mediated by the NART. There was no direct path from father's social class or mother's education to the ACE-III, but these had independent associations with childhood cognition, educational attainment and midlife occupational complexity, in descending order of magnitude. *APOE* ε4 showed a modest direct negative association with the ACE-III score, but was not associated with childhood cognition or the NART. When the model was re-run replacing *APOE* with the PGS, the path to the ACE-III was of negligible magnitude (β=0.004 95% CI -0.031, 0.038, p=0.82). The paths from the latter to childhood cognition and the ACE-III were also nonsignificant (β = -.02, 95% CI -0.05, 0.019, p = 0.35; β = 0.002, 95% CI -0.04, 0.04, p = 0.9, respectively). However, the path from PGS to NART was significantly negative (β = -0.03, 95% CI -0.06, -0.004, p = 0.03).

When the model was re-run on the ACE-III sub-scales, *APOE* ε4 was associated with Attention and Memory, with a similar magnitude to that of the total score (Supplementary Table 1); this reached 5% significance when these two scales were combined. Associations between *APOE* ε4 and the Language, Fluency and Visuospatial scales were negligible.

For peer review only

Childhood cognition was significantly associated with Attention, Memory and Fluency (negatively in the case of the latter, even though other variables were associated in the expected direction), but not Language or Visuospatial. Education, occupational complexity and the NART were associated with Fluency, Memory and Visuospatial to varying degrees, but none of these were significantly associated with Attention, and only occupational complexity was associated with Language (Supplementary Table 1).

## DISCUSSION

In the NSHD we estimated a path model describing key life course influences on cognitive state using the Addenbrooke's cognitive examination (ACE-III). Confirming our main study hypothesis, by far the strongest influence on this outcome was from lifetime cognition, most strongly from general cognitive ability in midlife, assessed by the NART. The NART in turn was particularly influenced by childhood cognition. To a lesser extent educational attainment was positively associated with the ACE-III, independently of childhood cognition, although the model suggests that this was part-mediated by the NART. Occupational complexity showed more modest effects still, and there were no direct associations between either measure of childhood SEP (mother's education and father's occupational social class) and the ACE-III, although these latter variables were associated with the intervening variables with magnitudes directly proportional to proximity. Finally, there was a direct negative association between the *APOE* ε4 allele and the ACE-III; ε4 was not associated with childhood cognitive function, nor with the NART. The pattern of associations for parental SEP, childhood cognition and education broadly reflect those previously shown in this cohort when the NART was an outcome rather than a predictor[2], even with an important genetic influence on cognitive function (APOE ε4) controlled. However, it is notable that, with the NART controlled, childhood cognition, education and midlife SEP additionally showed direct

For peer review only

associations with the ACE-III, with childhood cognition having the strongest effect, and midlife SEP the weakest.

Major strengths of this study are: 1. the use of a large representative population-based birth cohort; 2. an extensive and comprehensive measure of cognitive state (ACE-III) as an outcome; 3. prospective measures across the life course, including tested childhood cognition, which enabled a comprehensive prospective life course model of mental state; 4. path modelling that uses FIML parameter estimates for incomplete data, thus minimizing effects of missing predictor data. Against these strengths we should note the disproportionate loss to follow-up in those less socially advantaged, with lower prior cognitive function, and with higher physical morbidity. Also, the path structure of our model may be specific to the cohort (NSHD is ethnically homogenous) and period (NSHD experienced selective secondary education and high occupational mobility at labor market entry). While our previous work suggests a broadly robust path structure in the face of social change[12], replication in more diverse populations is required before our model can be considered generalizable.

Our path model suggests that cognitive state has a prominent general cognitive ability component, which in turn has cognitive antecedents extending back into childhood. It might be argued that the influence of the NART is a matter of circularity, reflecting the dominance of verbal-based tests within the ACE-III (accounting for 84% of the total score). However, the NART also correlates with non-verbal skills[9]. The most obvious difference between the NART and the ACE-III is that the constituent tests of the latter are ‘fluid’ measures, sensitive to age and morbidity-associated decline; whereas the former, as a measure of ‘crystallized’ ability, is stable even in the face of mild dementia[13]. Further follow-up will determine whether the cognitive paths within our model retain their magnitude and pattern as the ACE-III scores change over time.

For peer review only

In regard to the long-term cognitive antecedents of the ACE-III, the present study extends our previous studies showing that childhood cognition tracks across the life course even when education, lifetime socioeconomic position[2] and adolescent mental health[14] are controlled. This tracking is also consistent with earlier studies in relation to cognitive ageing[15] and risk of dementia[16,17]; and with studies showing that associations between tests of mental state and verbal cognitive ability are strongly explained by childhood cognitive function[18,19]. We also observed an additional direct association between childhood cognition and the ACE-III that was independent of the NART as well as other factors in the model. This is probably because the measures of childhood cognition capture a wider range of function than the NART, including nonverbal reasoning, even though, as noted, the NART itself predicts a comprehensive range of cognitive function[9].

The next most prominent influence on the ACE-III was from educational attainment, which was primarily based on qualifications through formal education, but also captured qualifications achieved up to early midlife, whether through job training or other paths through adult education. This was associated with the ACE-III even when childhood cognition was controlled. As with childhood cognition itself, the influence of education was largely through the NART, although again there was a modest independent association with the ACE-III, since education also shapes non-verbal cognitive skills[20]. An association between education and subsequent cognition independent of childhood cognition has long been observed[21]; has been replicated in two other birth cohorts[22]; is shown in NSHD to be additive with respect to adult education[23]; and responds rapidly to policy[12]. By way of interpretation, it is important to note that education is not just a process of 'cognitive stimulation'. Schools indeed teach specific knowledge, but can also teach practical skills, including how to approach cognitive testing, refine other cognitive skills, and shape non-cognitive skills that are likely to have long-term benefit to cognitive function[24,14]. Policies

For peer review only

to improve access to education, and widen educational curricula to strengthen all these skills, are likely to have long-term benefits to cognitive ageing, and risk of dementia.

Finally, we should consider the role of *APOE* in the model. This is involved in the transport of cholesterol and other lipids between cellular structures, and ε4 has a higher rate of lipoprotein clearance thus altering its bioavailability[25]. *APOE* is also involved in clearing beta amyloid from the brain, and ε4 may be less efficient at this[26]. A direct association between the ε4 allele and the ACE-III was found in our model; this was of relatively weak magnitude, was only observed in homozygotes, and was not observed with any other variable in the model including prior cognitive function. These findings are consistent with evidence that ε4 zygosity shows a dose-response for Alzheimer's disease[27]; with a study showing no association with childhood cognition although observed in old age in the same cohort[3]; and with parallel evidence from NSHD that decline in verbal memory from age 43 to 69 is faster in *APOE* homozygosity[28]. There is no consensus over whether *APOE* is associated with normal cognitive ageing as opposed to clinical decline[3,27-33]. However, this may be age-dependent[30]; intriguingly, while no association was found between ε4 and fluid cognitive measures in NSHD at age 53[28,31], this association is now evident 16 years later, albeit modestly. In regard to cognitive domain, it is interesting to note the finding that *APOE* ε4 was associated with attention and memory in particular. This is a potentially important finding since three of five neuropsychological tests identified by a meta-analysis as having the highest predictive accuracy for progression from mild cognitive impairment to Alzheimer's disease were of episodic memory[34]. It should also be highlighted that the presence of *APOE* in the model means that the structure and magnitude of the pathways, including those between parental social class and childhood cognition, were independent of this. Adding *APOE* does not of course comprehensively control for genetic influence on

For peer review only

cognitive ageing. However, the ε4 allele of this gene is the best-known genetic risk factor for clinically significant cognitive decline[35].

In contrast to *APOE* ε4, a polygenic score for AD was associated with a lower NART score, this was not associated with the ACE-III, although scores based on the same IGAP database used as a reference for the PRS in this study are predictive of AD itself[36]. The lack of association between a PGS for AD and general cognitive ability is consistent with a recent study using the Lothian birth cohort[37], although these authors found an association for cognitive slope (but not intercept) with a more stringent whole-genome threshold (0.01) than ours (0.05). They suggest that SNPs unrelated to *APOE* ε4 may be overpowering the signal from this; indeed, a systematic analysis of the GenAge database found *APOE* to be one of the top 3 genes associated with the greatest number of age-related diseases[38].

In conclusion, the ACE-III in the general ageing population shows a pattern of life course antecedents that is similar to neuropsychological measures of cognitive function. This may not have emerged from studies using briefer tests of cognitive state such as the MMSE, since most of these have ceiling effects outside the clinical context that limit their use as continuous measures. As noted, continuing follow-up of NSHD will elucidate whether the path structure we describe here changes as an increasing number of participants meet clinical criteria for dementia, and the distribution of the ACE-III shifts accordingly.

**Acknowledgements**

We thank NSHD study members for their lifelong participation and past and present members of the NSHD study team who helped to collect the data. We also thank ACE Mobile for providing a customized version of the ACE-III for NSHD.

For peer review only

**Funding**

This study was supported by the UK Medical Research Council, which provides core funding for the NSHD, and supports the authors by MC_UU_12019/1 (Enhancing NSHD), and /3 (Mental Ageing). DD is additionally funded through a Wellcome Trust Intermediate Clinical Fellowship (WT107467). SNJ is additionally funded by Alzheimer's Research UK (ARUK-PG2014–1946, ARUK-PG2017-1946).

**Data Sharing**

Bona fide researchers can apply to access the NSHD data via a standard application procedure (further details available at: http://www.nshd.mr. cac.uk/data.aspx).

**Competing interests**

None disclosed.

Figure legend: Figure 1. Path model for the ACE-III total score

For peer review only

## REFERENCES

1 Kuh D, Wong A, Shah I, et al. The MRC National Survey of Health and Development reaches age 70: maintaining participation at older ages in a birth cohort study. *Eur J Epidemiol* 2016;31:1135–1147.

2 Richards M, Sacker A. Lifetime antecedents of cognitive reserve. *J Clin Exp Neuropsychol* 2003;25:614–624.

3 Deary IJ, Whiteman MC, Pattie A, et al. Cognitive change and the APOE epsilon 4 allele. *Nature* 2002;418:932. (erratum Nature 2002;419:450)

4 Hsieh S, Schubert S, Hoon C, et al. Validation of the Addenbrooke's Cognitive Examination III in frontotemporal dementia and Alzheimer's disease. *Dement Geriatr Cogn Disord* 2013;36:242–250.

5 Lambert JC, Ibrahim-Verbaas CA, Harold D, et al. Meta-analysis of 74,046 individuals identifies 11 new susceptibility loci for Alzheimer's disease. *Nat Genet* 2013;45:1452-8.

6 Kaplan GA, Turrell G, Lynch JW, Everson SA, Helkala EL, Salonen JT. Childhood socioeconomic position and cognitive function in adulthood. *Int J Epidemiol* 2001;30:256-63.

7 Pigeon DA. Tests used in the 1954 and 1957 surveys. In: Douglas JWB ed. The home and the school. London: Macgibbon & Key 1964 appendix 1:129-132.

8 *ONS*. 2014. The National Statistics Socio-economic Classification (NS-SEC rebased on the SOC2010) [Online]. Newport, Wales: Office for National Statistics. Available: http://www.ons.gov.uk/ons/guide-method/classifications/current-standard-classifications/soc2010/soc2010-volume-3-ns-sec--rebased-on-soc2010--user-manual/index.html.

For peer review only

9 Nelson HE, Willison JR. National Adult Reading Test (NART). 2nd ed. Windsor: NFER-Nelson 1991.

10 *StataCorp*. 2017. Stata Statistical Software: Release 15. College Station, TX: StataCorp LLC

11 Arbuckle JC. Full information estimation in the presence of incomplete data. In: Marcoulides GA, Schumacker RE eds. Advanced Structural Equation Modeling Techniques. Mahwah, NJ: Lawrence Erlbaum Associates, 1996:243-277.

12 Richards M, Power C, Sacker A. Paths to literacy and numeracy problems: evidence from two British birth cohorts. *J Epidemiol Community Health* 2009;63:239–244.

13 McGurn B, Starr JM, Topfer JA, et al. Pronunciation of irregular words is preserved in dementia, validating premorbid IQ estimation. *Neurology* 2004;62:1184–1186.

14 Xu MK, Jones PB, Barnett JH, et al. Adolescent self-organization predicts midlife memory in a prospective birth cohort study. *Psychology Aging* 2013;28:958–68.

15 Plassman B, Welsh K, Helms M, et al. Intelligence and education as predictors of cognitive state in late life: A 50-year follow-up. *Neurology* 1995;45:1446–1450.

16 Snowdon DA, Kemper SJ, Mortimer JA, et al. Linguistic ability in early life and cognitive function and Alzheimer's disease in late life. Findings from the Nun Study. *JAMA* 1996;275:528–532.

17 McGurn B, Deary IJ, Starr JM. Childhood cognitive ability and risk of late-onset Alzheimer and vascular dementia. *Neurology* 2008;71:1051–1056.

18 Crawford JR, Deary IJ, Starr J, et al. The NART as an index of prior intellectual functioning: a retrospective validity study covering a 66-year interval. *Psychol Med* 2001;31:451–458.

For peer review only

19 Dykiert D, Der G, Starr JM, et al. Why is Mini-Mental state examination performance correlated with estimated premorbid cognitive ability? *Psychol Med* 2016;46:2647–2654.

20 Roselli R, Ardila A. The impact of culture and education on non-verbal neuropsychological measurements: A critical review. *Brain Cognition* 2003;52:326–333.

21 Snow RE, Yalow E. Education and intelligence. In: Sternberg RJ, ed. Handbook of human intelligence. Cambridge, MA: Cambridge University Press, 1982:493-585.

22 Clouston S, Kuh D, Herd P, et al. Benefits of educational attainment on adult fluid cognition: international evidence from three birth cohorts. *Int J Epidemiol* 2012;41:1729–1736.

23 Hatch SL, Feinstein L, Link B, et al. The continuing benefits of education: adult education and midlife cognitive ability in the British 1946 birth cohort. *J Gerontol Series B* 2007;62:S404–414.

24 Richards M, Hatch, SL. A life course approach to the development of mental skills. *Journal of Gerontology Series B* 2011;66:Suppl 1, i26–35.

25 Poirier J, Davignon J, Bouthillier D, et al. Apolipoprotein E polymorphism and Alzheimer's disease. *Lancet* 1993;342:697–699.

26 Jiang Q, Lee CY, Mandrekar S, et al. ApoE promotes the proteolytic degradation of Aβ. *Neuron* 2008;58:681–693.

27 Jorm AF, Mather KA, Butterworth P, et al. APOE genotype and cognitive functioning in a large age-stratified population sample. *Neuropsychology* 2007;21:1–8.

For peer review only

28 Rawle M, Davis D, Bendayan B, et al. Apolipoprotein-E (APOE) ε4 and cognitive decline over the adult life course. *Transl Psychiat* 2018 Jan 10;8:18. doi: 10.1038/s41398-017-0064-8.

29 Caselli RJ, Dueck AC, Osborne D, et al. Longitudinal Modeling of Age-Related Memory Decline and the APOE ε4 Effect. *N Engl J Med* 2009;361:255–263.

30 Wisdom NM, Callahan JL, Hawkins KA. The effects of apolipoprotein E on non-impaired cognitive functioning: a meta-analysis. *Neurobiol Aging* 2011;32:63–74.

31 Alfred T, Ben-Shlomo Y, Cooper R, et al. Associations between APOE and low-density lipoprotein cholesterol genotypes and cognitive and physical capability: the HALCyon programme. *Age* 2014;36:9673.

32 Bunce D, Bielak AA, Anstey KJ, et al. APOE genotype and cognitive change in young, middle-aged, and older adults living in the community. *J Gerontol A Biol Sci Med Sci* 2014;69:379–386.

33 Knight RG, Tsui HS, Abraham WC, et al. Lack of effect of the apolipoprotein E epsilon4 genotype on cognition during healthy aging. *J Clin Exp Neuropsychol* 2014:36:742–750.

34 Belleville S, Fouquet C, Hudon C, Zomahoun HTV, Consortium for the Early Indentification of Alzheimer's disease-Quebec. Neuropsychological measures that predict progression from mild cognitive impairment to Alzheimer's type dementia in older adults: a systematic review and meta-analysis. Neuropsychol Rev 2017;27:328-353.

35 Farrer LA, Cupples LA, Haines JL, et al. Effects of age, sex, and ethnicity on the association between apolipoprotein E genotype and Alzheimer disease. A meta-analysis. APOE and Alzheimer Disease Meta Analysis Consortium. *JAMA* 1997;278:1349–1356.

For peer review only

36 Escott-Price V, Sims R, Bannister C, et al. Common polygenic variation enhances risk prediction for Alzheimer's disease. *Brain* 2015;138:3673–3684.

37 Ritchie S, David Hill W, Riccardo E. Marioni RE, et al. Polygenic predictors of age-related decline in cognitive ability. *bioRxiv* Jul. 24, 2018;doi: http://dx.doi.org/10.1101/375691.

38 Fernandes M, Wan C, Tacutu R, et al. Systematic analysis of the gerontome reveals links between aging and age-related diseases. *Hum Mol Genet* 2016;25:4804-4818.

For peer review only

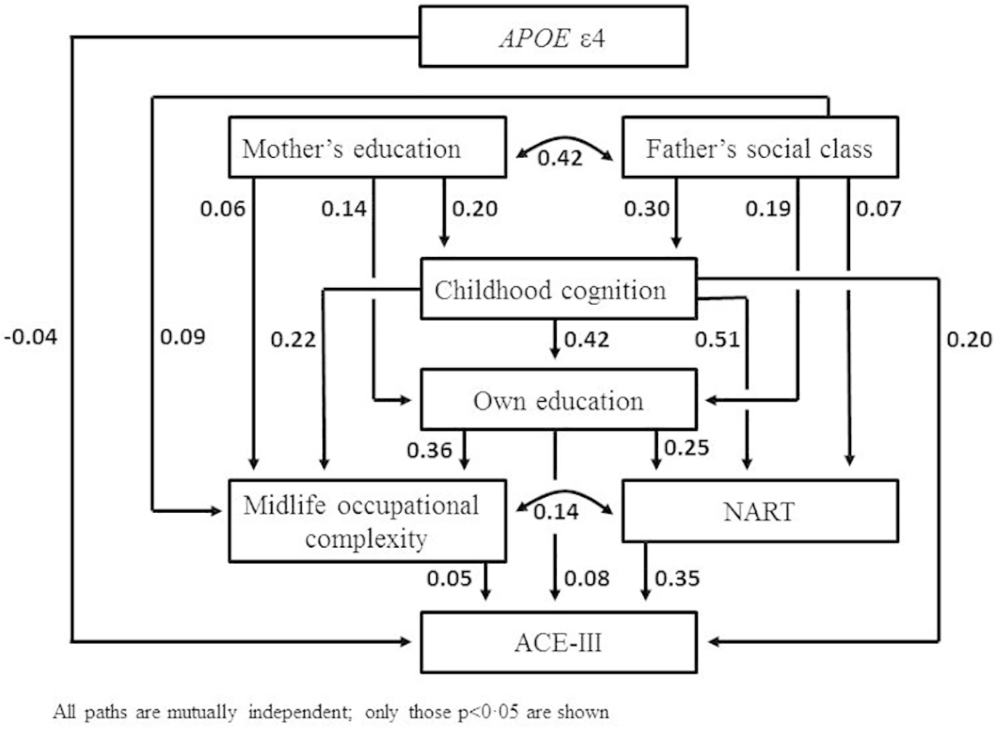


90x90mm (300 x 300 DPI)

**Supplementary Table 1** Associations between path variables and each ACE-III subscale: β (95% confidence intervals)

| | Attention | Memory | Language | Fluency | Visuospatial |
|---|---|---|---|---|---|
| *APOE* ε4[1] | -0.05 (-0.1, 0.003) | -0.03 (-0.08, 0.01) | 0.009 (-0.05, 0.06) | 0.0003 (-0.04, 0.04) | 0.003 (-0.05, 0.06) |
| Father's social class | -0.02 (-0.08, 0.03) | 0.04 (-0.01, 0.09) | 0.04 (-0.02, 0.09) | 0.02 (-0.02, 0.05)* | 0.04 (-0.02, 0.09) |
| Mother's education | 0.04 (-0.01, 0.09) | 0.01 (-0.03, 0.06) | -0.02 (-0.07, 0.03) | -0.007 (-0.04, 0.03) | -0.02 (-0.07, 0.04) |
| Childhood cognition | 0.12 (0.05, 0.20)*** | 0.10 (0.04, 0.17)*** | -0.01, -0.08, 0.06) | -0.09 (-0.13, -0.04)*** | -0.008 (-0.08, 0.06) |
| Education | 0.05 (-0.01, 0.12) | 0.07 (0.02, 0.13)* | 0.05 (-0.01, 0.12) | 0.09 (0.05, 0.13)*** | 0.06 (-0.001, 0.13) |
| NART | 0.04 (-0.03, 0.11) | 0.30 (0.23, 0.36)*** | 0.06 (-0.02, 0.14) | 0.19 (0.14, 0.25)*** | 0.04 (-0.04, 0.12) |
| Occupational complexity | 0.01 (-0.04, 0.07) | 0.05 (0.001, 0.10)* | 0.06 (0.005, 0.12)* | 0.08 (0.05, 0.12)*** | 0.06 (0.004, 0.12)* |

[1] -0.05 (-0.09, -0.002), $p = 0.04$ for combined Attention and Memory

* $p < 0.05$, *** $p < 0.001$

For peer review only

**STROBE 2007 (v4) Statement—Checklist of items that should be included in reports of *cohort studies***

**Checklist completed for: Developmental and adult risk factors associated with decline in grip strength from midlife to old age: a British birth cohort study**

| Section/Topic | Item # | Recommendation | Reported on page # |
|---|---|---|---|
| **Title and abstract** | 1 | (*a*) Indicate the study's design with a commonly used term in the title or the abstract | Title & abstract |
| | | (*b*) Provide in the abstract an informative and balanced summary of what was done and what was found | 2 |
| **Introduction** | | | |
| Background/rationale | 2 | Explain the scientific background and rationale for the investigation being reported | 4-5 |
| Objectives | 3 | State specific objectives, including any prespecified hypotheses | 5 |
| **Methods** | | | |
| Study design | 4 | Present key elements of study design early in the paper | 5-7 |
| Setting | 5 | Describe the setting, locations, and relevant dates, including periods of recruitment, exposure, follow-up, and data collection | 5-7 |
| Participants | 6 | (*a*) Give the eligibility criteria, and the sources and methods of selection of participants. Describe methods of follow-up | 5-7 |
| | | (*b*) For matched studies, give matching criteria and number of exposed and unexposed | n/a |
| Variables | 7 | Clearly define all outcomes, exposures, predictors, potential confounders, and effect modifiers. Give diagnostic criteria, if applicable | 6-7 |
| Data sources/ measurement | 8* | For each variable of interest, give sources of data and details of methods of assessment (measurement). Describe comparability of assessment methods if there is more than one group | *6-7* |
| Bias | 9 | Describe any efforts to address potential sources of bias | 9 |
| Study size | 10 | Explain how the study size was arrived at | 5 |
| Quantitative variables | 11 | Explain how quantitative variables were handled in the analyses. If applicable, describe which groupings were chosen and why | 6-7, 8-9 |
| Statistical methods | 12 | (*a*) Describe all statistical methods, including those used to control for confounding | 8-9 |
| | | (*b*) Describe any methods used to examine subgroups and interactions | 8-9 |
| | | (*c*) Explain how missing data were addressed | 6-7,9 |
| | | (*d*) If applicable, explain how loss to follow-up was addressed | 9 |
| | | (*e*) Describe any sensitivity analyses | 9 |

For peer review only

| | | | |
|---|---|---|---|
| **Results** | | | |
| Participants | 13* | (a) Report numbers of individuals at each stage of study—eg numbers potentially eligible, examined for eligibility, confirmed eligible, included in the study, completing follow-up, and analysed | 5-7, all tables |
| | | (b) Give reasons for non-participation at each stage | 5,16 |
| | | (c) Consider use of a flow diagram | - |
| Descriptive data | 14* | (a) Give characteristics of study participants (eg demographic, clinical, social) and information on exposures and potential confounders | 9, Table 1 |
| | | (b) Indicate number of participants with missing data for each variable of interest | Table 1 |
| | | (c) Summarise follow-up time (eg, average and total amount) | 6 |
| Outcome data | 15* | Report numbers of outcome events or summary measures over time | 6-12, all tables |
| Main results | 16 | (*a*) Give unadjusted estimates and, if applicable, confounder-adjusted estimates and their precision (eg, 95% confidence interval). Make clear which confounders were adjusted for and why they were included | 9-12, all tables |
| | | (*b*) Report category boundaries when continuous variables were categorized | All relevant tables |
| | | (*c*) If relevant, consider translating estimates of relative risk into absolute risk for a meaningful time period | - |
| Other analyses | 17 | Report other analyses done—eg analyses of subgroups and interactions, and sensitivity analyses | 12, all tables |
| **Discussion** | | | |
| Key results | 18 | Summarise key results with reference to study objectives | 12-13 |
| **Limitations** | | | |
| Interpretation | 20 | Give a cautious overall interpretation of results considering objectives, limitations, multiplicity of analyses, results from similar studies, and other relevant evidence | 14-15 |
| Generalisability | 21 | Discuss the generalisability (external validity) of the study results | 15-16 |
| **Other information** | | | |
| Funding | 22 | Give the source of funding and the role of the funders for the present study and, if applicable, for the original study on which the present article is based | 24 |

*Give information separately for cases and controls in case-control studies and, if applicable, for exposed and unexposed groups in cohort and cross-sectional studies.

**Note:** An Explanation and Elaboration article discusses each checklist item and gives methodological background and published examples of transparent reporting. The STROBE checklist is best used in conjunction with this article (freely available on the Web sites of PLoS Medicine at http://www.plosmedicine.org/, Annals of Internal Medicine at http://www.annals.org/, and Epidemiology at http://www.epidem.com/). Information on the STROBE Initiative is available at www.strobe-statement.org.

For peer review only

BMJ Open

# Identifying the lifetime cognitive and socioeconomic antecedents of cognitive state: seven decades of follow-up in a British birth cohort study.

| | |
|---|---|
| Journal: | *BMJ Open* |
| Manuscript ID | bmjopen-2018-024404.R2 |
| Article Type: | Research |
| Date Submitted by the Author: | 05-Feb-2019 |
| Complete List of Authors: | Richards, M; UCL, MRC Unit for Lifelong Health and Ageing at UCL<br>James, Sarah-Naomi; MRC Unit for Lifelong Health and Ageing<br>Sizer, Alison; University College London, Epidemiology and Public Health<br>Sharma, Nikhil; MRC Unit for Lifelong Health and Ageing; University College London Hospitals NHS Foundation Trust National Hospital for Neurology and Neurosurgery<br>Rawle, Mark; MRC Unit for Lifelong Health and Ageing<br>Davis, Daniel; MRC Unit for Lifelong Health and Ageing<br>Kuh, Diana; MRC Unit for Lifelong Health and Ageing, |
| <b>Primary Subject Heading</b>: | Epidemiology |
| Secondary Subject Heading: | Geriatric medicine |
| Keywords: | EPIDEMIOLOGY, Dementia < NEUROLOGY, Old age psychiatry < PSYCHIATRY |
| | |

SCHOLARONE™ Manuscripts

# Identifying the lifetime cognitive and socioeconomic antecedents of cognitive state: seven decades of follow-up in a British birth cohort study

Marcus Richards [1], Sarah-Naomi James [1], Alison Sizer [2], Nikhil Sharma [1,3], Mark James Rawle [1], Daniel Davis [1], Diana Kuh [1].

[1] MRC Unit for Lifelong Health and Ageing at UCL, 33 Bedford Place, London WC1B 5JU, UK

[2] Department of Epidemiology and Public Health, University College London, 1-19 Torrington Place, London, WC1E 7HB, UK

[3] National Hospital for Neurology and Neurosurgery, Queen Square, London WC1N 3BG, UK

**Correspondence to** Professor Marcus Richards, MRC Unit for Lifelong Health and Ageing at UCL, 33 Bedford Place, London WC1B 5JU, UK; m.richards@ucl.ac.uk tel. +44 (0)207 670 5710

Word count

Abstract: 266

Main text: 3509

Contributorship statement

MR conceived the work, conducted the statistical analyses and wrote the manuscript; SNJ advised on the path modelling; SNJ, AS, NS, MJR,, DD and DK contributed to interpretation and writing.

For peer review only

**ABSTRACT**

**Objectives** The life course determinants of midlife and later life cognitive function have been studied using longitudinal population-based cohort data, but far less is known about whether the pattern of these pathways is similar or distinct for clinically-relevant cognitive state. We investigated this for the Addenbrooke's Cognitive Examination (ACE-III), used in clinical settings to screen for cognitive impairment and dementia.

**Design** Longitudinal birth cohort study.

**Setting** Residential addresses in England, Wales and Scotland.

**Participants** 1762 community-dwelling men and women of European heritage, enrolled since birth in the MRC National Survey of Health and Development (the British 1946 birth cohort).

**Primary outcome** The Addenbrooke's Cognitive Examination (ACE-III).

**Results** Path modelling estimated direct and indirect associations between *APOE* status, father's social class, childhood cognition, education, midlife occupational complexity, midlife verbal ability (National Adult Reading Test; NART), and the total ACE-III score. Controlling for sex, there was a direct negative association between *APOE* ε4 and the ACE-III score (β=-0.04, [-0.08, -0.002], p=0.04), but not between *APOE* ε4 and childhood cognition (β=0.03 [-0.006, 0.69, p=0.10] or the NART (β=0.0005 [-0.03, 0.03], p=0.97). The strongest influences on the ACE-III were from childhood cognition (β=0.20 [0.14, 0.26], p<0.001) and the NART (β=0.35 [0.29, 0.41], p<0.001); educational attainment and

For peer review only

occupational complexity were modestly and independently associated with the ACE-III ($\beta$=0.08 [0.03, 0.14], p=0.002 and $\beta$=0.05 [0.01, 0.10], p=0.02, respectively).

**Conclusions** The ACE-III in the general population shows a pattern of life course antecedents that is similar to neuropsychological measures of cognitive function, and may be utilised to represent normal cognitive ageing as well as a screen for cognitive impairment and dementia.

For peer review only

**Strengths and limitations of this study**

- The MRC National Survey of Health and Development (the British 1946 birth cohort) is a large population-based sample with prospectively obtained information on socioeconomic status and educational attainment, and tested cognitive function from childhood
- The Addenbrooke's Cognitive Examination (ACE-III) is an extensive and comprehensive test of cognitive state.
- Path modelling used parameter estimates for incomplete data, thus minimising effects of missing predictor data
- The path structure of our model may be specific to cohort; NSHD is ethnically homogenous and experienced selective secondary education and high occupational mobility at labour market entry).
- Replication in more diverse populations is therefore required before our model can be considered generalisable

For peer review only

## INTRODUCTION

Using the MRC National Survey of Health and Development (NSHD; the British 1946 birth cohort)[1], we demonstrated multiple paths linking four fundamental developmental and social factors to midlife cognitive function: father's socioeconomic position (SEP), childhood cognitive ability, educational attainment, and own midlife SEP[2]. To our knowledge such a path model to understand key life course influences on cognitive state, as assessed in clinical practice, has not been undertaken. This is partly because the most frequently used tests, such as the Mini Mental Status Examination (MMSE) and Montreal Cognitive Assessment (MoCA), are brief and have pronounced ceiling effects. It would however be valuable to investigate whether life course paths to cognitive state show a similar pattern as those for other cognitive functions, which would inform theoretical understanding of, and methodology for, studies of cognitive ageing across the full population range. At the most recent NSHD wave at age 69, the Addenbrooke's Cognitive Examination 3rd edition (ACE-III) was administered. This is a more extensive and comprehensive test of cognitive state than the MMSE or MoCA, with a quasi-normal distribution. Using this outcome we estimated a path model incorporating childhood SEP, childhood cognitive ability, educational attainment and midlife occupational complexity, and adding two new paths. First, the National Adult Reading Test (NART), an outcome in the original path model, was now included as an intervening variable; we hypothesised that influences on cognitive state operate significantly through this test. Second, the apolipoprotein E (*APOE*) gene was included, the best known genetic risk factor for dementia; based on previous work[3] we hypothesised that the ε4 allele of this gene would be negatively associated with the ACE-III score but not with childhood cognition.

For peer review only - http://bmjopen.bmj.com/site/about/guidelines.xhtml

# METHODS

## Participants

The NSHD is a representative sample of 5362 males and females born in England, Scotland, and Wales in one week in March 1946 (http://www.nshd.mrc.ac.uk/nshd). The 24th data collection was conducted between 2014 and 2015 when study members were aged 68-69 years[1]. At age 69 study members still alive and with a known current address in mainland Britain (n=2698) were invited to have a home visit by a trained nurse; 2149 (79.7%) completed a visit and a further 55 (2·0%) completed a postal questionnaire instead. Of the original cohort, 1026 (19·1%) had died, 578 (10·8%) were living abroad, 22 (0·4%) asked for their participation to be restricted to postal contacts, 621 (11·6%) had previously withdrawn from the study, and 417 (7·8%) had been lost to follow-up. For this data collection we obtained ethical approval from the NRES Queen Square REC (14/LO/1073), and Scotland A REC (14/SS/1009). All participants gave written informed consent to collect these data.

## Measures

Principal outcome: the ACE -III

The ACE-III is a screen-implemented test of cognitive state, and has been validated as a screening tool for cognitive deficits in Alzheimer's disease and frontotemporal dementia[4]. The ACE-III is divided into five domains: attention & orientation (scored 0-18), verbal fluency (0-14), memory (0-26), language (0-26), and visuospatial function (0-16). Thus the maximum total score is 100. Due to the inclusion of verbal fluency, the distribution of the total score is quasi-normal and avoids the pronounced ceiling effect of most cognitive state tests. A customised version of the ACE-III was administered by iPad using ACEMobile (http://www.acemobile.org/); where this was not possible, a paper version was used. All offline scoring was undertaken by trained personnel. Of the 2149 participants who had a

For peer review only

home visit, 32 refused or were unable to undertake the ACE-III. Of the remaining 2117, 35 undertook but did not complete this; and for the remaining 2082, data for 320 were lost through equipment failure. Thus complete ACE-III data were available for 1762 participants, 82·0% of those who received a home visit.

Genetic risk

Genetic risk was primarily represented by the *APOE* ε4 allele. Using blood taken at age 53 or 69-71 by a research nurse, KBioscience analysed SNPs rs429358 and rs7412 to determine *APOE* genotype. Distribution of alleles was as follows (n = 2686), ε2/ε2 n=20 (0·74%), ε2/ε3 n=318 (11·84%), ε3/ε3 n=1538 (57·26%), ε2/ε4 n=68 (2·53%), ε3/ε4 n=657 (24·46%), ε4/ε4 n=85 (3·16%). For analysis, *APOE* genotype was recoded categorically for the presence of ε4 alleles, with carriers of ε2 included as non *APOE* ε4 carriers. Because of difficulties in interpreting potentially opposing effects on cognition, the 68 participants with ε2/ε4 were excluded. Thus *APOE* was categorized as no ε4 vs. heterozygous ε4 or homozygous ε4. For comparison with *APOE*, polygenic scores (PGS) for Alzheimer's disease were calculated for 2,768 participants using blood samples taken at age 53 and 60-64. Genotyping was carried out on the NeuroX2 chip. PGSs were created using Allelic Scoring function in PLINK_v1.9. The base dataset used to calculate the PGS was the large, two-stage meta-analysis of genome-wide association studies in individuals of European heritage conducted by the International Genomics of Alzheimer's Project (IGAP)[5]. Linkage-disequilibrium parameters were set to $r2>0.2$ and a physical distance threshold for clumping SNPs set to 1Mb. The PGS included the SNPs with a p-value in the I-GAP meta-analysis of $p<0.05$ (n= 31746).

Early life SEP

Early life SEP was assessed using father's occupational social class and mother's education, which is associated with offspring cognition independently of father's occupation[6].   The

For peer review only

former was classified when participants were aged 11 (or at 4 or 15 years if this was unknown) according to the UK Registrar General: professional, managerial, intermediate, skilled manual, semiskilled manual, unskilled; mother's education was coded as primary only vs. secondary or any formal qualifications.

Childhood cognitive function

At 8 years participants took tests of verbal and nonverbal ability devised by the National Foundation for Educational Research[7], and administered by teachers or other trained personnel. These tests were: (1) reading comprehension (selecting appropriate words to complete 35 sentences), (2) word reading (ability to pronounce 50 words), (3) vocabulary (ability to explain the meaning of these 50 words), and (4) picture intelligence, consisting of a 60-item nonverbal reasoning test. Scores for each test were standardized to the whole sample, then summed to create a total score representing overall cognitive ability at this age.

Educational attainment

The highest educational qualification achieved by 43 years was grouped into no qualification, below ordinary secondary qualifications (vocational), ordinary secondary qualifications ('O' levels and their training equivalents), advanced secondary qualifications ('A' levels and their equivalents), or higher qualifications (degree or equivalent).

Midlife occupational complexity

Midlife occupational complexity was represented by the National Statistics Socio-Economic Classification (NS-SEC) of the job held at age 53 or earlier if this was missing[8]. This provides a measure of employment relations and the conditions of employment, based on the Standard Occupational Classification (SOC): details of individual employment status (i.e.

For peer review only

employer/ employee/ self-employed); supervisory position; and number of employees in the workplace. For NSHD information was available on the start and end dates of up to 27 jobs and their NS-SEC categories, which were recoded into 7 classes: 1. Higher managerial, administrative and professional occupations; 2. Lower managerial, administrative and professional occupations; 3. Intermediate occupations; 4. Small employers and own account workers; 5. Lower supervisory and technical occupations; 6. Semi-routine occupations; 7. Routine occupations.

The NART

The NART assesses ability to pronounce 50 words of increasing difficulty[9]. These words violate conventional pronunciation rules, and are therefore unlikely to be read correctly unless the reader is familiar with them rather than relies on intelligent guesswork. Thus the NART serves as a measure of general (crystallised') cognitive ability.

All measures were coded so that higher values signified higher status or function.

**Statistical methods**

All statistical analyses were conducted using STATA version 15[10]. Path modelling was used to quantify associations between each predictor variable and the ACE-III. Since each of the predictor variables are closely inter-related, the model also quantifed their independent inter-associations. We hypothesized two key components within this model: 1. strong paths from childhood cognition and the NART to the ACE-III, with modest and weak additional contributions from education and midlife occupational complexity, respectively, and no direct path from childhood SEP[2]; 2. a direct negative path from APOE ε4 to the ACE-III but not via childhood cognition[3] or the NART. No directionality of association was

For peer review only

assumed between mother's education and father's social class, or between occupational complexity and the NART; these paths are therefore represented as correlational only. The path model was adjusted for gender, andincorporated full information maximum likelihood (FIML) parameter estimates to include those with item-missingness. FIML is preferable to estimation based on complete data, since FIML estimates tend to be less biased and more reliable than estimates based on list-wise deletion, even when the data deviate from missing at random and are non-ignorable[11]. Three criteria were used to assess model fit: 1. the $\chi 2$ test, although this can be overly sensitive to model misspecification when sample sizes are large; 2. the root mean square error of approximation (RMSEA), which gives a measure of the discrepancy in fit per degrees of freedom. It is bounded below by zero, only taking this value if the model fits exactly. If the RMSEA is $< 0.05$, the model is considered a close fit to the data; 3. the comparative fit index (CFI), whose values are restricted to a 0 to 1 continuum, with higher values indicating a better fit. CFI is normally tested against a minimum criterion value of 0.95.

**Patient and Public Involvement**

Participants have a lifelong association with NSHD. Over the 70 years of the study, the research team has increasingly involved participants, in line with changing norms about conducting cohort studies, starting at age 16 (in 1962) with the annual dissemination of study findings in birthday cards and this continues. Participants have always received a personal letter from the Director whenever they have raised queries or provided additional comments, including suggestions for new topics to study. In the last ten years, the research team has increased the level of participant involvement through invitations to study events and focus groups to discuss clinical sub-studies; and a new participant website (www.nshd.mrc.ac.uk/study-members/) was developed in line with their feedback. When

For peer review only

piloting new questionnaires and assessments, we recruit patients from GP practices or from the UCLH PPI and take into account their feedback when designing the mainstage fieldwork for NSHD participants.

## RESULTS

### Descriptive

As noted, sample size was 1762, the maximum N for the ACE-III. Those who were not interviewed at age 69 for any reason showed no difference in *APOE* ε4 frequency (p=0.72) but had lower childhood cognition and NART scores, and were more likely to be disadvantaged in terms of father's social class, mother's education, own education and occupational complexity (all p<0.001). Those not interviewed were also previously shown to have three or more clinical disorders at the previous assessment (age 60-64), a general health self-rating as poor or fair rather than good, and a longstanding limiting illness, although the latter was not associated with interview participation after controlling for socioeconomic and cognitive characteristics[1]. Of those interviewed at age 69, there were no differences in any of the path variables between those with and without ACE-III data, except for a slight trend for the ACE-III to be missing in those with no educational qualifications ($\chi 2$=9.5, p= 0.05). Frequencies for each category of APOE group, childhood and midlife SEP and educational attainment, and means and SDs for the ACE-III and NART, are shown in Table 1.

**Table 1** Frequency distributions for APOE group, childhood and midlife SEP, educational attainment, and mean NART and ACE-III scores (for 1762 participants with ACE-III data)

| Variable | N | % | N | % |
|---|---|---|---|---|

| | | |
|---|---|---|
| *APOE* | | |
| No ε4 | 1076 | (61·1) |
| Heterozygous ε4 | 388 | (21·0) |
| Homozygous ε4 | 48 | (2·7) |
| Missing | 250 | (14·2) |
| | | |
| Father's social class | | |
| Professional | 134 | (7·6) |
| Managerial | 372 | (21·1) |
| Intermediate | 296 | (16·8) |
| Skilled manual | 519 | (29·4) |
| Semiskilled | 271 | (15·4) |
| Unskilled | 79 | (4·5) |
| Missing | 91 | (5·2) |
| | | |
| Mother's education | | |
| Primary only | 1151 | (65.3) |
| Secondary or any | | |
| formal qualifications | 421 | (23.9) |
| Missing | 611 | (10.8) |
| | | |
| Educational attainment (by age 43) | | |
| No qualifications | 403 | (22.9) |
| Vocational only | 235 | (13·3) |

For peer review only

| | | |
|---|---|---|
| Ordinary ('O') level | 340 | (19.3) |
| Advanced ('A') level | 497 | (28.2) |
| Higher | 2.73 | (15.5) |
| Missing | 14 | (0.8) |
| | | |
| NSSEC occupation (by age 53)* | | |
| 1. | 221 | (12.5) |
| 2. | 504 | (28.6) |
| 3. | 307 | (17.4) |
| 4. | 200 | (11.4) |
| 5. | 120 | (6.8) |
| 6. | 228 | (12.9) |
| 7. | 154 | (8.7) |
| Missing | 28 | (1.6) |
| | | |
| | Mean | (SE) |
| NART | 35.6 | (0.22) |
| ACE-III | 91.52 | (0.14) |

*1. Higher managerial, administrative and professional occupations; 2. Lower managerial, administrative and professional occupations; 3. Intermediate occupations; 4. Small employers and own account workers; 5. Lower supervisory and technical occupations; 6. Semi-routine occupations; 7. Routine occupations.

For peer review only

**Path model**

Figure 1 shows the path model. All paths are mutually adjusted. Goodness of fit statistics indicated that the model was an excellent representation of the data ($\chi^2$=0.15, p=1.0 for analytic vs. saturated model; RMSEA=0, p=1·0; CFI = 1·0). Gender effects and all non-significant paths (*P* value >.05) are not shown.

[Figure 1 about here]

The strongest influences on the ACE-III score were from the NART, and from childhood cognition, which was mainly associated with the ACE-III via educational attainment and the NART, but also directly with the ACE-III. The influence of midlife occupational complexity was more modest, and was itself part-mediated by the NART. There was no direct path from father's social class or mother's education to the ACE-III, but these had independent associations with childhood cognition, educational attainment and midlife occupational complexity, in descending order of magnitude. *APOE* ε4 showed a modest direct negative association with the ACE-III score, but was not associated with childhood cognition or the NART. When the model was re-run replacing *APOE* with the PGS, the path to the ACE-III was of negligible magnitude (β=0.004 95% CI -0.031, 0.038, p=0.82). The paths from the latter to childhood cognition and the ACE-III were also nonsignificant (β = -.02, 95% CI -0.05, 0.019, p = 0.35; β = 0.002, 95% CI -0.04, 0.04, p = 0.9, respectively). However, the path from PGS to NART was significantly negative (β = -0.03, 95% CI -0.06, -0.004, p = 0.03).

When the model was re-run on the ACE-III sub-scales, *APOE* ε4 was associated with Attention and Memory, with a similar magnitude to that of the total score (Supplementary Table 1); this reached 5% significance when these two scales were combined. Associations between *APOE* ε4 and the Language, Fluency and Visuospatial scales were negligible.

For peer review only - http://bmjopen.bmj.com/site/about/guidelines.xhtml

Childhood cognition was significantly associated with Attention, Memory and Fluency (negatively in the case of the latter, even though other variables were associated in the expected direction), but not Language or Visuospatial. Education, occupational complexity and the NART were associated with Fluency, Memory and Visuospatial to varying degrees, but none of these were significantly associated with Attention, and only occupational complexity was associated with Language (Supplementary Table 1).

## DISCUSSION

In the NSHD we estimated a path model describing key life course influences on cognitive state using the Addenbrooke's cognitive examination (ACE-III). Confirming our main study hypothesis, by far the strongest influence on this outcome was from lifetime cognition, most strongly from general cognitive ability in midlife, assessed by the NART. The NART in turn was particularly influenced by childhood cognition. To a lesser extent educational attainment was positively associated with the ACE-III, independently of childhood cognition, although the model suggests that this was part-mediated by the NART. Occupational complexity showed more modest effects still, and there were no direct associations between either measure of childhood SEP (mother's education and father's occupational social class) and the ACE-III, although these latter variables were associated with the intervening variables with magnitudes directly proportional to proximity. Finally, there was a direct negative association between the *APOE* ε4 allele and the ACE-III; ε4 was not associated with childhood cognitive function, nor with the NART. The pattern of associations for parental SEP, childhood cognition and education broadly reflect those previously shown in this cohort when the NART was an outcome rather than a predictor[2], even with an important genetic influence on cognitive function (APOE ε4) controlled. However, it is notable that, with the NART controlled, childhood cognition, education and midlife SEP additionally showed direct

For peer review only

associations with the ACE-III, with childhood cognition having the strongest effect, and midlife SEP the weakest.

Major strengths of this study are: 1. the use of a large representative population-based birth cohort; 2. an extensive and comprehensive measure of cognitive state (ACE-III) as an outcome; 3. prospective measures across the life course, including tested childhood cognition, which enabled a comprehensive prospective life course model of mental state; 4. path modelling that uses FIML parameter estimates for incomplete data, thus minimizing effects of missing predictor data. Against these strengths we should note the disproportionate loss to follow-up in those less socially advantaged, with lower prior cognitive function, and with higher physical morbidity. Also, the path structure of our model may be specific to the cohort (NSHD is ethnically homogenous) and period (NSHD experienced selective secondary education and high occupational mobility at labor market entry). While our previous work suggests a broadly robust path structure in the face of social change[12], replication in more diverse populations is required before our model can be considered generalizable.

Our path model suggests that cognitive state has a prominent general cognitive ability component, which in turn has cognitive antecedents extending back into childhood. It might be argued that the influence of the NART is a matter of circularity, reflecting the dominance of verbal-based tests within the ACE-III (accounting for 84% of the total score). However, the NART also correlates with non-verbal skills[9]. The most obvious difference between the NART and the ACE-III is that the constituent tests of the latter are 'fluid' measures, sensitive to age and morbidity-associated decline; whereas the former, as a measure of 'crystallized' ability, is stable even in the face of mild dementia[13]. Further follow-up will determine whether the cognitive paths within our model retain their magnitude and pattern as the ACE-III scores change over time. In this context it is important to note that the present study has

For peer review only

not yet incorporated the clinical outcomes of Mild Cognitive Impairment (MCI) and dementia, where a life course approach to the latter has been described[14]. Cognitive decline from approximately the same age has been observed elsewhere when participants with these outcomes were excluded[15]; and also across midlife in NSHD, not explained by concomitant medical conditions[16], the treatment of which can increase risk of MCI and dementia[17,18].

In regard to the long-term cognitive antecedents of the ACE-III, the present study extends our previous studies showing that childhood cognition tracks across the life course even when education, lifetime socioeconomic position[2] and adolescent mental health[19] are controlled. This tracking is also consistent with earlier studies in relation to cognitive ageing[20] and risk of dementia[21,22]; and with studies showing that associations between tests of mental state and verbal cognitive ability are strongly explained by childhood cognitive function[23,24]. We also observed an additional direct association between childhood cognition and the ACE-III that was independent of the NART as well as other factors in the model. This is probably because the measures of childhood cognition capture a wider range of function than the NART, including nonverbal reasoning, even though, as noted, the NART itself predicts a comprehensive range of cognitive function[9].

The next most prominent influence on the ACE-III was from educational attainment, which was primarily based on qualifications through formal education, but also captured qualifications achieved up to early midlife, whether through job training or other paths through adult education. This was associated with the ACE-III even when childhood cognition was controlled. As with childhood cognition itself, the influence of education was largely through the NART, although again there was a modest independent association with the ACE-III, since education also shapes non-verbal cognitive skills[25]. An association between education and subsequent cognition independent of childhood cognition has long

For peer review only

been observed[26]; has been replicated in two other birth cohorts[27]; is shown in NSHD to be additive with respect to adult education[28]; and responds rapidly to policy[12]. By way of interpretation, it is important to note that education is not just a process of 'cognitive stimulation'. Schools indeed teach specific knowledge, but can also teach practical skills, including how to approach cognitive testing, refine other cognitive skills, and shape non-cognitive skills that are likely to have long-term benefit to cognitive function[29,19]. Policies to improve access to education, and widen educational curricula to strengthen all these skills, are likely to have long-term benefits to cognitive ageing, and risk of dementia.

Finally, we should consider the role of *APOE* in the model. This is involved in the transport of cholesterol and other lipids between cellular structures, and ε4 has a higher rate of lipoprotein clearance thus altering its bioavailability[30]. *APOE* is also involved in clearing beta amyloid from the brain, and ε4 may be less efficient at this[31]. A direct association between the ε4 allele and the ACE-III was found in our model; this was of relatively weak magnitude, was only observed in homozygotes, and was not observed with any other variable in the model including prior cognitive function. These findings are consistent with evidence that ε4 zygosity shows a dose-response for Alzheimer's disease[32]; with a study showing no association with childhood cognition although observed in old age in the same cohort[3]; and with parallel evidence from NSHD that decline in verbal memory from age 43 to 69 is faster in *APOE* homozygosity[33]. There is no consensus over whether *APOE* is associated with normal cognitive ageing as opposed to clinical decline[3,32-38]. However, this may be age-dependent[35]; intriguingly, while no association was found between ε4 and fluid cognitive measures in NSHD at age 53[33,36], this association is now evident 16 years later, albeit modestly. In regard to cognitive domain, it is interesting to note the finding that *APOE* ε4 was associated with attention and memory in particular. This is a potentially important finding since three of five neuropsychological tests identified by a meta-analysis as having

For peer review only

the highest predictive accuracy for progression from mild cognitive impairment to Alzheimer's disease were of episodic memory[39]. It should also be highlighted that the presence of *APOE* in the model means that the structure and magnitude of the pathways, including those between parental social class and childhood cognition, were independent of this. Adding *APOE* does not of course comprehensively control for genetic influence on cognitive ageing. However, the ε4 allele of this gene is the best-known genetic risk factor for clinically significant cognitive decline[40].

In contrast to *APOE* ε4, a polygenic score for AD was associated with a lower NART score, this was not associated with the ACE-III, although scores based on the same IGAP database used as a reference for the PRS in this study are predictive of AD itself[41]. The lack of association between a PGS for AD and general cognitive ability is consistent with a recent study using the Lothian birth cohort[42], although these authors found an association for cognitive slope (but not intercept) with a more stringent whole-genome threshold (0.01) than ours (0.05). They suggest that SNPs unrelated to *APOE* ε4 may be overpowering the signal from this; indeed, a systematic analysis of the GenAge database found *APOE* to be one of the top 3 genes associated with the greatest number of age-related diseases[43].

In conclusion, the ACE-III in the general ageing population shows a pattern of life course antecedents that is similar to neuropsychological measures of cognitive function. This may not have emerged from studies using briefer tests of cognitive state such as the MMSE, since most of these have ceiling effects outside the clinical context that limit their use as continuous measures. As noted, continuing follow-up of NSHD will elucidate whether the path structure we describe here changes as an increasing number of participants meet clinical criteria for dementia, and the distribution of the ACE-III shifts accordingly.

**Acknowledgements**

For peer review only

We thank NSHD study members for their lifelong participation and past and present members of the NSHD study team who helped to collect the data. We also thank ACE Mobile for providing a customized version of the ACE-III for NSHD.

**Funding**

This study was supported by the UK Medical Research Council, which provides core funding for the NSHD, and supports the authors by MC_UU_12019/1 (Enhancing NSHD), and /3 (Mental Ageing). DD is additionally funded through a Wellcome Trust Intermediate Clinical Fellowship (WT107467). SNJ is additionally funded by Alzheimer's Research UK (ARUK-PG2014–1946, ARUK-PG2017-1946).

**Data Sharing**

Bona fide researchers can apply to access the NSHD data via a standard application procedure (further details available at: http://www.nshd.mr. cac.uk/data.aspx).

**Competing interests**

None disclosed.

Figure legend: Figure 1. Path model for the ACE-III total score

For peer review only

## REFERENCES


1 Kuh D, Wong A, Shah I, et al. The MRC National Survey of Health and Development reaches age 70: maintaining participation at older ages in a birth cohort study. *Eur J Epidemiol* 2016;31:1135–1147.

2 Richards M, Sacker A. Lifetime antecedents of cognitive reserve. *J Clin Exp Neuropsychol* 2003;25:614–624.

3 Deary IJ, Whiteman MC, Pattie A, et al. Cognitive change and the APOE epsilon 4 allele. *Nature* 2002;418:932. (erratum Nature 2002;419:450)

4 Hsieh S, Schubert S, Hoon C, et al. Validation of the Addenbrooke's Cognitive Examination III in frontotemporal dementia and Alzheimer's disease. *Dement Geriatr Cogn Disord* 2013;36:242–250.

5 Lambert JC, Ibrahim-Verbaas CA, Harold D, et al. Meta-analysis of 74,046 individuals identifies 11 new susceptibility loci for Alzheimer's disease. *Nat Genet* 2013;45:1452-8.

6 Kaplan GA, Turrell G, Lynch JW, Everson SA, Helkala EL, Salonen JT. Childhood socioeconomic position and cognitive function in adulthood. *Int J Epidemiol* 2001;30:256-63.

7 Pigeon DA. Tests used in the 1954 and 1957 surveys. In: Douglas JWB ed. The home and the school. London: Macgibbon & Key 1964 appendix 1:129-132.

8 *ONS*. 2014. The National Statistics Socio-economic Classification (NS-SEC rebased on the SOC2010) [Online]. Newport, Wales: Office for National Statistics. Available: http://www.ons.gov.uk/ons/guide-method/classifications/current-standard-classifications/soc2010/soc2010-volume-3-ns-sec--rebased-on-soc2010--user-manual/index.html.


For peer review only

9 Nelson HE, Willison JR. National Adult Reading Test (NART). 2nd ed. Windsor: NFER-Nelson 1991.

10 *StataCorp*. 2017. Stata Statistical Software: Release 15. College Station, TX: StataCorp LLC.

11 Arbuckle JC. Full information estimation in the presence of incomplete data. In: Marcoulides GA, Schumacker RE eds. Advanced Structural Equation Modeling Techniques. Mahwah, NJ: Lawrence Erlbaum Associates, 1996:243-277.

12 Richards M, Power C, Sacker A. Paths to literacy and numeracy problems: evidence from two British birth cohorts. *J Epidemiol Community Health* 2009;63:239–244.

13 McGurn B, Starr JM, Topfer JA, et al. Pronunciation of irregular words is preserved in dementia, validating premorbid IQ estimation. *Neurology* 2004;62:1184–1186.

14 Whalley LJ1, Dick FD, McNeill G. A life-course approach to the aetiology of late-onset dementias. *Lancet Neurol* 2006;5:87-96.

15 McCarrey AC, An Y, Kitner-Triolo MH, Ferrucci L, Resnick SM. Sex differences in cognitive trajectories in clinically normal older adults. Psychol Aging 2016;31:166-175.

16 Davis D, Cooper R, Muniz Terrera G, Hardy R, Richards M, Kuh D. Verbal memory and search speed in early midlife are associated with mortality over 25 years follow-up, independently of health status and early life factors. A British birth cohort study. *Int J Epidemiol* 2016;45:1216-1225.

17 Rawle MJ, Cooper R, Kuh D, Richards M. Associations between polypharmacy and cognitive and physical capability: A British Birth Cohort Study. *J Am Geriatr Soc* 2018;66:916-923.

For peer review only

18 Richardson K, Fox C, Maidment I, et al. Anticholinergic drugs and risk of dementia: case-control study. *BMJ* 2018;361:k1315.

19 Xu MK, Jones PB, Barnett JH, et al. Adolescent self-organization predicts midlife memory in a prospective birth cohort study. *Psychology Aging* 2013;28:958–68.

20 Plassman B, Welsh K, Helms M, et al. Intelligence and education as predictors of cognitive state in late life: A 50-year follow-up. *Neurology* 1995;45:1446–1450.

21 Snowdon DA, Kemper SJ, Mortimer JA, et al. Linguistic ability in early life and cognitive function and Alzheimer's disease in late life. Findings from the Nun Study. *JAMA* 1996;275:528–532.

22 McGurn B, Deary IJ, Starr JM. Childhood cognitive ability and risk of late-onset Alzheimer and vascular dementia. *Neurology* 2008;71:1051–1056.

23 Crawford JR, Deary IJ, Starr J, et al. The NART as an index of prior intellectual functioning: a retrospective validity study covering a 66-year interval. *Psychol Med* 2001;31:451–458.

24 Dykiert D, Der G, Starr JM, et al. Why is Mini-Mental state examination performance correlated with estimated premorbid cognitive ability? *Psychol Med* 2016;46:2647–2654.

25 Roselli R, Ardila A. The impact of culture and education on non-verbal neuropsychological measurements: A critical review. *Brain Cognition* 2003;52:326–333.

26 Snow RE, Yalow E. Education and intelligence. In: Sternberg RJ, ed. Handbook of human intelligence. Cambridge, MA: Cambridge University Press, 1982:493-585.

27 Clouston S, Kuh D, Herd P, et al. Benefits of educational attainment on adult fluid cognition: international evidence from three birth cohorts. *Int J Epidemiol* 2012;41:1729–1736.

For peer review only

28 Hatch SL, Feinstein L, Link B, et al. The continuing benefits of education: adult education and midlife cognitive ability in the British 1946 birth cohort. *J Gerontol Series B* 2007;62:S404–414.

29 Richards M, Hatch, SL. A life course approach to the development of mental skills. *Journal of Gerontology Series B* 2011;66:Suppl 1, i26–35.

30 Poirier J, Davignon J, Bouthillier D, et al. Apolipoprotein E polymorphism and Alzheimer's disease. *Lancet* 1993;342:697–699.

31 Jiang Q, Lee CY, Mandrekar S, et al. ApoE promotes the proteolytic degradation of Aβ. *Neuron* 2008;58:681–693.

32 Jorm AF, Mather KA, Butterworth P, et al. APOE genotype and cognitive functioning in a large age-stratified population sample. *Neuropsychology* 2007;21:1–8.

33 Rawle M, Davis D, Bendayan B, et al. Apolipoprotein-E (APOE) ε4 and cognitive decline over the adult life course. *Transl Psychiat* 2018 Jan 10;8:18. doi: 10.1038/s41398-017-0064-8.

34 Caselli RJ, Dueck AC, Osborne D, et al. Longitudinal Modeling of Age-Related Memory Decline and the APOE ε4 Effect. *N Engl J Med* 2009;361:255–263.

35 Wisdom NM, Callahan JL, Hawkins KA. The effects of apolipoprotein E on non-impaired cognitive functioning: a meta-analysis. *Neurobiol Aging* 2011;32:63–74.

36 Alfred T, Ben-Shlomo Y, Cooper R, et al. Associations between APOE and low-density lipoprotein cholesterol genotypes and cognitive and physical capability: the HALCyon programme. *Age* 2014;36:9673.

37 Bunce D, Bielak AA, Anstey KJ, et al. APOE genotype and cognitive change in young, middle-aged, and older adults living in the community. *J Gerontol A Biol Sci Med Sci* 2014;69:379–386.

For peer review only

38 Knight RG, Tsui HS, Abraham WC, et al. Lack of effect of the apolipoprotein E epsilon4 genotype on cognition during healthy aging. *J Clin Exp Neuropsychol* 2014:36:742–750.

39 Belleville S, Fouquet C, Hudon C, Zomahoun HTV, Consortium for the Early Indentification of Alzheimer's disease-Quebec. Neuropsychological measures that predict progression from mild cognitive impairment to Alzheimer's type dementia in older adults: a systematic review and meta-analysis. Neuropsychol Rev 2017;27:328-353.

40 Farrer LA, Cupples LA, Haines JL, et al. Effects of age, sex, and ethnicity on the association between apolipoprotein E genotype and Alzheimer disease. A meta-analysis. APOE and Alzheimer Disease Meta Analysis Consortium. *JAMA* 1997;278:1349–1356.

41 Escott-Price V, Sims R, Bannister C, et al. Common polygenic variation enhances risk prediction for Alzheimer's disease. *Brain* 2015;138:3673–3684.

42 Ritchie S, David Hill W, Riccardo E. Marioni RE, et al. Polygenic predictors of age-related decline in cognitive ability. *bioRxiv* Jul. 24, 2018;doi: http://dx.doi.org/10.1101/375691.

43 Fernandes M, Wan C, Tacutu R, et al. Systematic analysis of the gerontome reveals links between aging and age-related diseases. *Hum Mol Genet* 2016;25:4804-4818.

For peer review only

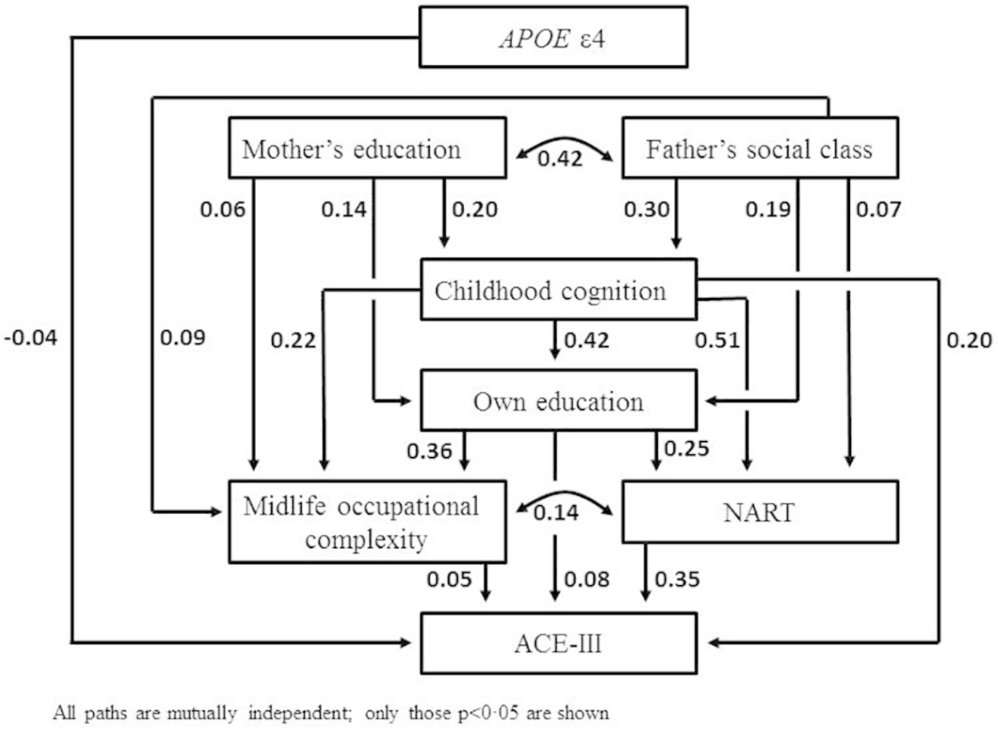


90x90mm (300 x 300 DPI)

**Supplementary Table 1** Associations between path variables and each ACE-III subscale: β (95% confidence intervals)

| | Attention | Memory | Language | Fluency | Visuospatial |
|---|---|---|---|---|---|
| *APOE* ε4[1] | -0.05 (-0.1, 0.003) | -0.03 (-0.08, 0.01) | 0.009 (-0.05, 0.06) | 0.0003 (-0.04, 0.04) | 0.003 (-0.05, 0.06) |
| Father's social class | -0.02 (-0.08, 0.03) | 0.04 (-0.01, 0.09) | 0.04 (-0.02, 0.09) | 0.02 (-0.02, 0.05)* | 0.04 (-0.02, 0.09) |
| Mother's education | 0.04 (-0.01, 0.09) | 0.01 (-0.03, 0.06) | -0.02 (-0.07, 0.03) | -0.007 (-0.04, 0.03) | -0.02 (-0.07, 0.04) |
| Childhood cognition | 0.12 (0.05, 0.20)*** | 0.10 (0.04, 0.17)*** | -0.01, -0.08, 0.06) | -0.09 (-0.13, -0.04)*** | -0.008 (-0.08, 0.06) |
| Education | 0.05 (-0.01, 0.12) | 0.07 (0.02, 0.13)* | 0.05 (-0.01, 0.12) | 0.09 (0.05, 0.13)*** | 0.06 (-0.001, 0.13) |
| NART | 0.04 (-0.03, 0.11) | 0.30 (0.23, 0.36)*** | 0.06 (-0.02, 0.14) | 0.19 (0.14, 0.25)*** | 0.04 (-0.04, 0.12) |
| Occupational complexity | 0.01 (-0.04, 0.07) | 0.05 (0.001, 0.10)* | 0.06 (0.005, 0.12)* | 0.08 (0.05, 0.12)*** | 0.06 (0.004, 0.12)* |

[1] -0.05 (-0.09, -0.002), p = 0.04 for combined Attention and Memory

* $p < 0.05$, *** $p < 0.001$

For peer review only

**STROBE 2007 (v4) Statement—Checklist of items that should be included in reports of *cohort studies***

**Checklist completed for: Developmental and adult risk factors associated with decline in grip strength from midlife to old age: a British birth cohort study**

| **Section/Topic** | **Item #** | **Recommendation** | **Reported on page #** |
|---|---|---|---|
| **Title and abstract** | 1 | (*a*) Indicate the study's design with a commonly used term in the title or the abstract | Title & abstract |
| | | (*b*) Provide in the abstract an informative and balanced summary of what was done and what was found | 2 |
| **Introduction** | | | |
| Background/rationale | 2 | Explain the scientific background and rationale for the investigation being reported | 4-5 |
| Objectives | 3 | State specific objectives, including any prespecified hypotheses | 5 |
| **Methods** | | | |
| Study design | 4 | Present key elements of study design early in the paper | 5-7 |
| Setting | 5 | Describe the setting, locations, and relevant dates, including periods of recruitment, exposure, follow-up, and data collection | 5-7 |
| Participants | 6 | (*a*) Give the eligibility criteria, and the sources and methods of selection of participants. Describe methods of follow-up | 5-7 |
| | | (*b*) For matched studies, give matching criteria and number of exposed and unexposed | n/a |
| Variables | 7 | Clearly define all outcomes, exposures, predictors, potential confounders, and effect modifiers. Give diagnostic criteria, if applicable | 6-7 |
| Data sources/ measurement | 8* | For each variable of interest, give sources of data and details of methods of assessment (measurement). Describe comparability of assessment methods if there is more than one group | *6-7* |
| Bias | 9 | Describe any efforts to address potential sources of bias | 9 |
| Study size | 10 | Explain how the study size was arrived at | 5 |
| Quantitative variables | 11 | Explain how quantitative variables were handled in the analyses. If applicable, describe which groupings were chosen and why | 6-7, 8-9 |
| Statistical methods | 12 | (*a*) Describe all statistical methods, including those used to control for confounding | 8-9 |
| | | (*b*) Describe any methods used to examine subgroups and interactions | 8-9 |
| | | (*c*) Explain how missing data were addressed | 6-7,9 |
| | | (*d*) If applicable, explain how loss to follow-up was addressed | 9 |
| | | (*e*) Describe any sensitivity analyses | 9 |

For peer review only

| | | | |
|---|---|---|---|
| **Results** | | | |
| Participants | 13* | (a) Report numbers of individuals at each stage of study—eg numbers potentially eligible, examined for eligibility, confirmed eligible, included in the study, completing follow-up, and analysed | 5-7, all tables |
| | | (b) Give reasons for non-participation at each stage | 5,16 |
| | | (c) Consider use of a flow diagram | - |
| Descriptive data | 14* | (a) Give characteristics of study participants (eg demographic, clinical, social) and information on exposures and potential confounders | 9, Table 1 |
| | | (b) Indicate number of participants with missing data for each variable of interest | Table 1 |
| | | (c) Summarise follow-up time (eg, average and total amount) | 6 |
| Outcome data | 15* | Report numbers of outcome events or summary measures over time | 6-12, all tables |
| Main results | 16 | (*a*) Give unadjusted estimates and, if applicable, confounder-adjusted estimates and their precision (eg, 95% confidence interval). Make clear which confounders were adjusted for and why they were included | 9-12, all tables |
| | | (*b*) Report category boundaries when continuous variables were categorized | All relevant tables |
| | | (*c*) If relevant, consider translating estimates of relative risk into absolute risk for a meaningful time period | - |
| Other analyses | 17 | Report other analyses done—eg analyses of subgroups and interactions, and sensitivity analyses | 12, all tables |
| **Discussion** | | | |
| Key results | 18 | Summarise key results with reference to study objectives | 12-13 |
| **Limitations** | | | |
| Interpretation | 20 | Give a cautious overall interpretation of results considering objectives, limitations, multiplicity of analyses, results from similar studies, and other relevant evidence | 14-15 |
| Generalisability | 21 | Discuss the generalisability (external validity) of the study results | 15-16 |
| **Other information** | | | |
| Funding | 22 | Give the source of funding and the role of the funders for the present study and, if applicable, for the original study on which the present article is based | 24 |

*Give information separately for cases and controls in case-control studies and, if applicable, for exposed and unexposed groups in cohort and cross-sectional studies.

**Note:** An Explanation and Elaboration article discusses each checklist item and gives methodological background and published examples of transparent reporting. The STROBE checklist is best used in conjunction with this article (freely available on the Web sites of PLoS Medicine at http://www.plosmedicine.org/, Annals of Internal Medicine at http://www.annals.org/, and Epidemiology at http://www.epidem.com/). Information on the STROBE Initiative is available at www.strobe-statement.org.

For peer review only
